# Predictive value of quantitative ^18^F-FDG-PET radiomics analysis in patients with head and neck squamous cell carcinoma

**DOI:** 10.1186/s13550-020-00686-2

**Published:** 2020-09-07

**Authors:** Roland M. Martens, Thomas Koopman, Daniel P. Noij, Elisabeth Pfaehler, Caroline Übelhör, Sughandi Sharma, Marije R. Vergeer, C. René Leemans, Otto S. Hoekstra, Maqsood Yaqub, Gerben J. Zwezerijnen, Martijn W. Heymans, Carel F. W. Peeters, Remco de Bree, Pim de Graaf, Jonas A. Castelijns, Ronald Boellaard

**Affiliations:** 1 Department of Radiology and Nuclear Medicine, Amsterdam University Medical Center, De Boelelaan 1117, PO Box 7057, 1007 Amsterdam, MB Netherlands; 2 Department of Nuclear Medicine and Molecular Imaging, Medical Imaging Center, University of Groningen, University Medical Center Groningen, Groningen, The Netherlands; 3 Department of Epidemiology and Biostatistics, Amsterdam University Medical Center, De Boelelaan, 1117 Amsterdam, Netherlands; 4 Department of Radiation Oncology, Amsterdam University Medical Center, De Boelelaan, 1117 Amsterdam, Netherlands; 5 Department of Otolaryngology-Head and Neck Surgery, Amsterdam University Medical Center, De Boelelaan, 1117 Amsterdam, Netherlands; 6 Department of Head and Neck Surgical Oncology, University Medical Center Utrecht, Utrecht, the Netherlands

**Keywords:** Head and Neck Neoplasms, Positron Emission Tomography Computed Tomography, Radiomics, Prognosis

## Abstract

**Abstract:**

**Background:**

Radiomics is aimed at image-based tumor phenotyping, enabling application within clinical-decision-support-systems to improve diagnostic accuracy and allow for personalized treatment. The purpose was to identify predictive 18-fluor-fluoro-2-deoxyglucose (^18^F-FDG) positron-emission tomography (PET) radiomic features to predict recurrence, distant metastasis, and overall survival in patients with head and neck squamous cell carcinoma treated with chemoradiotherapy.

**Methods:**

Between 2012 and 2018, 103 retrospectively (training cohort) and 71 consecutively included patients (validation cohort) underwent ^18^F-FDG-PET/CT imaging. The 434 extracted radiomic features were subjected, after redundancy filtering, to a projection resulting in outcome-independent meta-features (factors). Correlations between clinical, first-order ^18^F-FDG-PET parameters (e.g., SUVmean), and factors were assessed. Factors were combined with ^18^F-FDG-PET and clinical parameters in a multivariable survival regression and validated. A clinically applicable risk-stratification was constructed for patients’ outcome.

**Results:**

Based on 124 retained radiomic features from 103 patients, 8 factors were constructed. Recurrence prediction was significantly most accurate by combining HPV-status, SUVmean, SUVpeak, factor 3 (histogram gradient and long-run-low-grey-level-emphasis), factor 4 (volume-difference, coarseness, and grey-level-non-uniformity), and factor 6 (histogram variation coefficient) (CI = 0.645). Distant metastasis prediction was most accurate assessing metabolic-active tumor volume (MATV)(CI = 0.627). Overall survival prediction was most accurate using HPV-status, SUVmean, SUVmax, factor 1 (least-axis-length, non-uniformity, high-dependence-of-high grey-levels), and factor 5 (aspherity, major-axis-length, inversed-compactness and, inversed-flatness) (CI = 0.764).

**Conclusions:**

Combining HPV-status, first-order ^18^F-FDG-PET parameters, and complementary radiomic factors was most accurate for time-to-event prediction. Predictive phenotype-specific tumor characteristics and interactions might be captured and retained using radiomic factors, which allows for personalized risk stratification and optimizing personalized cancer care.

**Trial registration:**

Trial NL3946 (NTR4111), local ethics commission reference: Prediction 2013.191 and 2016.498. Registered 7 August 2013, https://www.trialregister.nl/trial/3946

## Statement of translational relevance

The current study provided new insights in image-based tumor phenotyping by assessing associations of primary tumor and lymphnode metastasis characteristics, as a basis for future research. The combination of clinical, first-order, and radiomics features showed complementary predictive value for locoregional recurrence, metastasis and overall survival, while maintaining predictive underlying processes. A clinical applicable risk stratification was presented to stratify patients, which might improve clinical-decision-support-systems and enhances patient-specific treatment efficacy.

## Introduction

Personalized cancer care of locally advanced head and neck squamous cell carcinoma (HNSCC) implies customization of therapy to the individual patient. This might improve the current overall 5-year survival rate of 50% (35–65%) [1]. Radiotherapy with or without chemotherapy is frequently applied but fails in 50% of the cases. In the vast majority (about 90%), the locoregional failure occurs within the first 2 years after treatment [2, 3]. The consequence of recurrent cancer is that surgical salvage therapy is generally the only option with curative intent, but this is associated with high morbidity [4]. More efficient pre-treatment response prediction may result in patient-tailored escalation or toxicity-reducing de-escalation (e.g., in radiosensitive HPV-positive patients) of (chemo)radiotherapy or a switch to different treatment options (e.g., surgery). Imaging is crucial in management because of its value on fast and non-invasive tumor staging, response monitoring, and prognosis prediction [5]. Exploration of quantitative imaging features might reflect underlying phenotype and response and thus may maximize the success of tailored treatments [6].

Radiomics focuses on the methodology of extensive image-based tumor phenotyping [7]. With radiomics, it may be possible to characterize phenotypic differences providing information on the whole-lesion microenvironment and surrounding area accounting for spatial and temporal heterogeneity, such as cellular morphology, proliferative capacity, metabolism, motility, angiogenic and oxygenation status, gene expression (including expression of cell surface markers, growth factor, and hormonal receptors), proliferative, immunogenic, and metastatic potential [5, 6, 8]. These characteristics might be captured by radiomics-derived tumor features (i.e., intensity, shape, or texture) and might be of complementary value to other clinical parameters to predict their effect on the chemo-radiosensitivity (i.e., quantity of tumoral radiosensitive cancer stem cells, the hypoxic fraction, reoxygenation of the tumor vicinity, and/or repopulation capacity throughout the course of therapy) [7, 9–11].

Radiomic features of functional imaging may provide additional information to anatomical imaging, because it provides information on pathophysiologic tumor characteristics [12, 13]. Positron-emission tomography (PET)/computed tomography (CT) using ^18^F-fluoro-deoxy-glucose (^18^F-FDG) measures tumoral metabolic activity and can be quantified with ^18^F-FDG-PET/CT by the standard uptake value (SUV). Pretreatment ^18^F-FDG-PET/CT was reported to be useful for detection, treatment decision support [14], planning [15, 16], and the prediction and detection of recurrences and long-term outcome [2]. PET-radiomics was superior over a CT-based model (CI_PET_ = 0.77 versus CI_CT_ = 0.72) [17] and might improve lesion characterization and patient outcome prediction compared to first-order PET parameters in daily clinical routine [18–21].

Identified radiomic associations give insight in the biological basis of imaging appearance and could aid targeted treatment decision-making and predict prognosis non-invasively. Radiomics was mainly analyzed in CT [22], or PET-CT separately [8, 10], but when combined with clinical features, it resulted in higher predictive and prognostic value [17, 23]. To our knowledge, a comparison of prediction models in head and neck with FDG-PET radiomic factors, SUV measurements (e.g., maximum or peak SUV), and clinical parameters, associated with patient’s outcome has not yet been described.

The aim of this study was to construct a model based on ^18^F-FDG-PET radiomics features to predict locoregional recurrence, distant metastasis, and overall survival (OS) in patients with locally advanced head and neck squamous cell carcinoma treated with chemoradiotherapy.

## Methods

### Data selection

Between 2012 and 2014, 103 patients were included retrospectively in our training cohort. Between 2014 and 2018, 81 consecutive patients were included independently from the training cohort in a validation cohort. These training and validation single-center cohorts were approved by the local institutional ethics committee (Amsterdam UMC Medisch Ethische ToetsingCommissie (METC), reference: 2013.191). A written informed consent was waived for the training cohort (reference: 2016.498), whereas for the validation cohort a written informed consent was obtained from all patients. Previously untreated patients with histologically proven HNSCC were included who were planned for chemoradiotherapy with curative intent (see Table 1). Exclusion criteria were nasopharyngeal tumors, age < 18 and pregnancy, previous locoregional treatment of HNSCC, or insufficient image quality. Within 5 weeks after baseline imaging, treatment was initiated consisting of a pre-determined regimen of chemoradiotherapy (CRT) during a period of 7 weeks; 70 Gy in 35 fractions with concomitant cisplatin (100 mg/m^2^ on days 1, 22, and 43 of radiotherapy)) or cetuximab (400 mg/m^2^ loading dose followed by seven weekly infusions of 250 mg/m^2^). Tobacco use was defined as a smoking history of ≥ 10 pack years. Alcohol use was defined as drinking 3 or more alcoholic drinks per day [24, 25]. Locoregional recurrence was defined as the location of primary tumor (PT) and/or lymph node metastases (LN). Locoregional failure was measured from the end of CRT to the date of local or regional histological proven relapse. Metastasis was defined as a distant location from the locoregional PT and LN. Overall survival time was measured from the end of CRT until a HNSCC-related death. These patient outcomes concerned locoregional recurrence, metastasis or death within 2 years of follow-up time or a minimal follow-up time of 2 years after the end of treatment.

**Table 1:** Patient characteristics

	Training cohort	Validation cohort
	Number (%)	Number (%)
Patients total	103	71
No of male patients	76 (73.8%)	53 (75.7%)
Age, years (mean, IQR)	62.3 (57.3–67.8)	63.3 (57.8–69.3)
Mean radiation dose, Gy	70	70
Chemotherapy		
Cisplatin	88 (85.4%)	57 (80%)
Cetuximab	15 (14.6%)	14 (20%)
T-stage		
2	46 (44.7%)	25 (35.2%)
3	24 (23.3%)	19 (26.8%)
4	33 (32%)	27 (38%)
N-stage		
0	14 (13.6%)	11 (15.5%)
1	13 (12.6%)	15 (21.1%)
2	75 (72.8%)	45 (63.4%)
3	1 (1%)	0 (0%)
HPV-status		
Positive	39 (37.9%)	26 (36.6%)
Negative	64 (62.1%)	45 (63.4%)
Tumor site		
Oropharynx	74 (71.8%)	51 (71.8%)
Hypopharynx	29 (28.2%)	20 (28.2%)
Overall alcohol history score (SD)	1.91 (1.19)	1.72 (1.24)
Smoking pack years, (IQR)	22.7 (18.2–38.9)	23.5 (19.3–41.3)
Follow-up time (mean, IQR)	31.5 (20.7–44.5)	26.4 (19.8–34.1)
Recurrence	27 (26.2%)	19 (27.1%)
Metastasis	10 (9.7%)	18 (25.7%)
Death	37 (35.9%)	22 (31.4%)
IQR: interquartile range		

### ^18^F-FDG-PET/CT acquisition

^18^F-FDG-PET/low-dose-CT was performed according to the EANM guidelines 1.0 and since 2015 using version 2.0 on a Gemini-TF or Ingenuity TF PET/CT (Philips Medical Systems, Best, The Netherlands) with EARL accreditation [26]. The examination was performed after a 6-h fasting period and adequate hydration. Scans with arms down were acquired; from mid-thigh to skull vertex, 60 min after intravenous administration of 2.5 MBq/kg ^18^F-FDG (3 min per bed position). The ^18^F-FDG-PET/CT images were reconstructed using time of flight iterative ordered subsets expectation maximization (3 iterations and 21 subsets) with photon attenuation correction using a low dose CT [27]. Reconstructed images of both PET scanners were acquired with similar settings and had an image matrix size of 144 × 144, voxel size of 4 × 4 × 4 mm, FWHM of 6.75 mm. Low-dose-CT was collected using a beam current of 50 mAs at 120 kV for anatomical correlation of 18F-FDG uptake and attenuation correction. CT-scans were reconstructed using an image matrix size of 512 × 512 resulting in pixel sizes of 1.17 × 1.17 mm and a slice thickness of 5 mm.

### Whole-lesion delineation

Whole-lesion delineation was performed, as previously described [28], by an experienced nuclear medicine physician with 5 years of experience (BZ) supervised by another nuclear medicine physician with 30 years of experience (OH) in head and neck nuclear medicine, respectively, with knowledge of the HNSCC diagnosis, TNM-stage (7th edition [29]), and primary tumor location for delineation of proven malignant lesions. Delineation of primary tumors (PT) was performed semi-automatically on ^18^F-FDG-PET/CT using a 50% isocontour of the SUVpeak of the tumor volume adapted for the local background, providing low variability, low number of outliers, and high repeatability [30, 31]. SUV was normalized to body weight. Within the volume of interest (VOI), the maximum and mean SUV were defined (SUVmax and SUVmean). SUVpeak was defined as the uptake in a 1-mL spherical VOI with the highest value across all tumor voxel locations. Partial volume effects were minimized by taking lesion only with a minimum volume of 4.2 mL into account (i.e., 3 times the PET system’s spatial resolution of 6.75 mm FWHM) [32].

### Feature extraction

Radiomic features were extracted from the FDG-PET images using the in-house built Accurate tool (for making vois) in combination with the RadCat tool for feature calculation (Supplement 10), as described previously [33–35]. It provides 3D implementation of feature extraction methods for four types of features: shape, intensity, texture based on co-occurrence, and run-length matrices (description of tumor voxels with homogeneous/heterogeneous high or low grey-levels) according to the International biomarker standardization initiative (IBSI) standard [36]. For each patient, 434 ^18^F-FDG-PET radiomics features were extracted. For the texture analysis, PET images were discretized to a fixed bin size of 0.25 SUV [34]. The radiomic features were not normalized and only raw values were used that were directly computed from the DICOM images. The radiomic data processing consisted of dimension reduction to arrive at a limited number of latent features that retain most of the information contained in the original feature-space (see the next subsection and Supplement 1).

### Radiomic data processing

#### Redundancy filtering

First, the marginal associations between the retained radiomic features of the patient in the retrospective *training cohort* were assessed in a heat map. As radiomic data are inherently multicollinear, some redundancy was expected: that is, there were pairs of features whose marginal correlation neared (negative) unity. Hence, redundancy filtering was performed, using a custom redundancy-filtering algorithm [37]. This algorithm removes the minimal number of features under a marginal correlation threshold, which we set at 0.95.

#### Correlation matrix regularization

The correlation matrix between the remaining features after redundancy filtering was ill-conditioned [38]. The remaining correlation matrix was subjected to ridge-regularization [38]. The optimal value of the penalty-parameter was determined by 5-fold cross-validation of the log-likelihood. We considered the scaled features (centered around 0 and variance 1) to avoid a situation where the features with the largest scale dominate the analysis.

#### Factor analytic data compression

Then, we performed a maximum likelihood factor analysis on the regularized feature-correlation matrix [38]. The goal was to reduce the dimension of the data without losing (much) information. When the features naturally clustered into latent factors (meta-features), it was desirable to extract these factors, as it allowed us to build a parsimonious model that retained (as much as possible) the information of the full feature set. A latent radiomic meta-feature represents a projection of the shared information in a collection of observed features. It represents a latent domain underlying a cluster of observables. The dimension of the latent space was determined by Guttman bounds [39]. The factor-solution was rotated to a simple (i.e., sparse) orthogonal structure.

#### Obtaining factor scores

After projection of the original variable-space onto the lower-dimensional factor-space, we desired factor scores: the score each individual obtains on each of the latent factors. These were obtained by regressing the latent features on the observed data by way of the obtained factor solution. The resulting factor scores of the retrospective training set were used as predictors in further modeling.

#### Validation

Previously described four steps were then performed separately in the prospective validation cohort in order to validate similar radiomic factors in the prediction analysis.

### Statistical analysis

The correlation between clinical parameters, standard ^18^F-FDG-PET/CT parameters (SUVmax, SUVmean, SUVpeak), and radiomic factors was determined in the *training and validation set* with Spearman’s correlation coefficient. Corresponding *p* values were multiplicity-corrected using Bonferroni’s method. The difference in outcome was assessed between patients who received cisplatin and cetuximab (log rank test). The difference in outcome was assessed for patients with a oropharyngeal and hypopharyngeal tumor location between HPV-positive and HPV-negative status (log rank test).

The prognostic performance of clinical parameters, ^18^F-FDG-PET/CT parameters, and radiomic factors was firstly assessed in the *training set* separately for the patient outcomes (locoregional recurrence, distant metastases, and death) by performing a Cox regression analysis. Thereafter, significant clinical, ^18^F-FDG-PET/CT parameters, and radiomic factors were combined in a multivariable analysis. Multivariable regression analysis was performed according to the TRIPOD-statement (Supplement 9), accepting *p* values up to 0.157 to enhance the model applicability to other patient groups [40, 41]. Predictive performance of the models was assessed by a 5-fold cross-validation [42] and by using the incident area under the receiver operating curves (ROC) and concordance index (CI).

The predictive accuracy of the constructed prediction models in the training set was validated in a separate *validation set*. The prognostic performance was assessed by the incident area under the receiver operating curves (ROC) and concordance index (CI). Finally, the prediction models were compared in the validation set using the log-likelihood chi-square test and area under the curve (AUC).

A risk calculator for all outcomes was constructed, based on the normalized standard hazard and the coefficient of each parameter or radiomic factor of the predictive model. This risk stratification was divided into a high (≥ 66%), medium (≥ 33–66%), and low risk (< 33%) for a patient outcome using the most accurate prediction model. The correlation assessment was performed on IBM SPSS Statistics for Windows. Analyses regarding the factor-analytical data-compression and prognostic modeling were performed with *R*.

## Results

### Patient characteristics

Overall, 184 patients were included, of which 103 retrospectively (training set) and 71 consecutive independent patients (validation set)(see table 1 for patient characteristics). The mean age of the training cohort was 62.3 years (inter-quartile range (IQR): 57.3–67.8). The mean age of the validation cohort was 63.3 (IQR 57.8–69.3). Treatment of all included patients consisted of pre-determined regimens: in 88 patients radiotherapy was combined with a cisplatin dose, 15 patients received radiotherapy with cetuximab. The mean follow-up time in the training set was 31.5 months (IQR: 20.7-44.5) and in the validation set 26.4 months (IQR 19.8–34.1). In the training cohort, 27 recurrences, 10 metastases, and 37 deaths occurred. In the validation cohort, 19 recurrences, 18 metastases, and 22 deaths occurred. The outcome was not significantly different between patients who received cisplatin and those who received cetuximab in the training set and test set; for recurrence (*p* = 0.071, *p* = 0.877, respectively), metastasis (*p* = 0.60, *p* = 0.295, respectively), and OS (*p* = 0.053, *p* = 0.276, respectively). The median OS in the training set for patients with cisplatin 32.1 months and for cetuximab 27.6 months and in the validation set for cisplatin 23.2 months and for cetuximab 18.1 months. A significant better OS was found for HPV-positive cancers with both oropharyngeal and hypopharyngeal primary tumor location (both *p* < 0.05).

### Radiomic factors

**Fig. 1:**
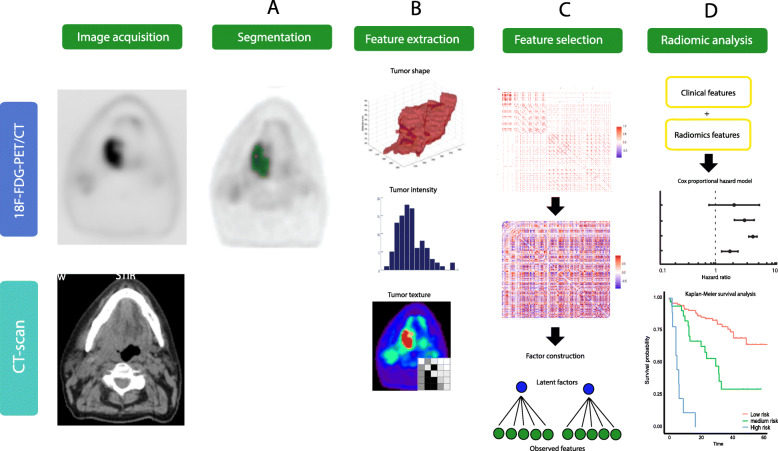
An overview of the radiomics workflow including **a** delineation, **b** extracting of intensity, texture, morphologic, and shape radiomics features. **c** The removal of redundancy of highly correlated features (Pearson *r* > 0.95) and the construction of factors. **d** The construction of prediction models with clinical, first-order PET-features, and/or radiomic factors and the risk-stratification into a high/medium/low risk for developing an event based on the constructed prediction models

Redundancy filtering showed many strong (absolute) associations, which was echoed in the heatmap on the thresholded correlation matrix (Fig. 1c), including all correlations whose absolute value equals or exceeds 0.95. After redundancy thresholding, 124 radiomic features were retained (Fig. 1d). The remaining correlation matrix was subjected to ridge-regularization with the optimal regularization parameter value determined by 5-fold cross-validation of the log-likelihood. The resulting regularized matrix was well-conditioned.

**Fig. 2:**
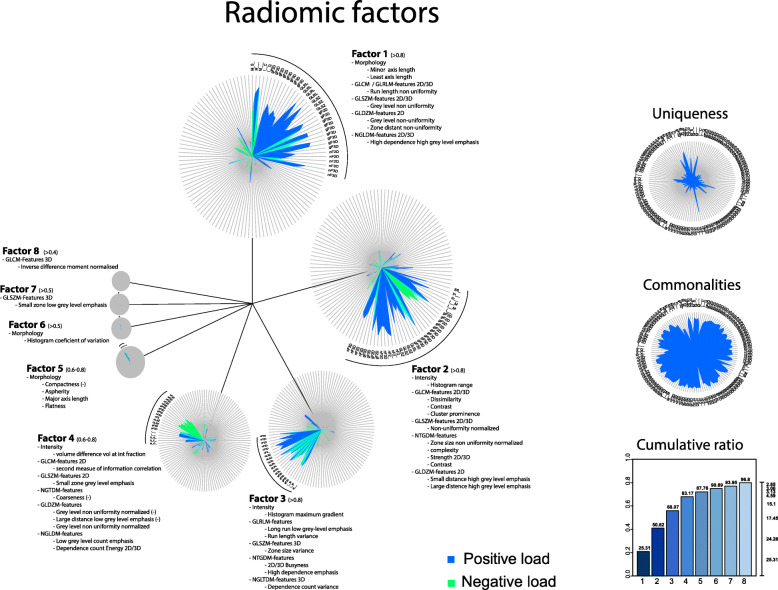
Dandelion plot for visualization of the dimension reduction of all features by construction of 8 factors reflecting the radiomics feature spectrum. The cumulative ratio was 80% of all extracted features. Per factor the most important radiomics features were described

The factor analytic data compression of the regularized correlation matrix resulted in eight latent meta-features (factors). These retained 80% of the covariation between the original 124 features. Hence, the factor solution was deemed to sufficiently represent the original feature-space (Supplement 1). The factor solution was visualized (Fig. 2) with a dandelion plot [43].

### Representation of original features in the radiomic factors

Factor 1 consisted mainly of (I) least axis length (morphology) and (II) non-uniformity (GLRLM; grey-level-run-length matrix and GLDZM; grey-level-distance zone-matrix (counts the number of groups of linked voxels, which share a specific discretized grey-level and possess the same distance to ROI edge), and (III) high dependence of high grey levels (NGLDM; neighborhood grey-level difference matrix, which aims to capture the coarseness of the overall texture [36]).

Factor 2 consisted mainly of (I) histogram range (intensity), (II) (A) contrast, dissimilarity, cluster prominence (GLCM; grey-level-co-occurrence matrix), (B) zone size non-uniformity (GLSZM; grey-level-size-zone matrix) (C) complexity, contrast, and strength (NTGDM; neighbourhood-grey-tone-difference matrices), and (D) small distance high grey level emphasis (GLDZM).

Factor 3 consisted mainly of (I) maximum histogram gradient and inversed minimum histogram gradient (Intensity), (II) (A) long run low grey-level emphasis and run-length variance (GLRLM), (B) zone size variance (GLSZM) (C) busyness (NGTDM), and (D) high dependence emphasis and dependence count variance (NGLDM).

Factor 4 consisted mainly of (I) volume difference (intensity), (II) (A) inversed 3D coarseness, grey-level non-uniformity, large distance low grey-level (NGTDM), and (B) inversed low grey-level count and energy count (NGLDM).

Factor 5 consisted mainly of (I) aspherity, major axis length, inversed compactness, and flatness (morphology).

Factor 6 consisted mainly of (I) histogram coefficient of variation (intensity) (II) second measure of information correlation (GLCM) and (III) Morans I (Morphology).

Factor 7 consisted mainly of (I) inversed small zone low grey-level emphasis (GLSZM).

Factor 8 consisted mainly of inversed difference features (GLCM), but scored lower than the overlapping factor 1 features.

### Associations between clinical and ^18^F-FDG-PET parameters with radiomic factors

The significant associations after Bonferroni’s correction of each of the 8 factors with T-stage, N-stage, HPV-status, and smoking in the training set (Table 2) showed that factor 1 had a significant positive correlation with T-stage (*r* = 0.454), SUVmax (*r* = 0.440), SUVpeak (*r* = 0.521), SUVmean (*r* = 0.468), TLG (*r* = 0.807), and MATV (*r* = 0.947). Factor 2 correlated significantly with SUVmax, SUVpeak, and SUVmean (*r* = 0.704–0.740). Furthermore, T-stage correlated significantly with SUVmax (*r* = 0.412), SUVpeak (*r* = 0.438), SUVmean (*r* = 0.422), and MATV (*r* = 0.405). HPV-status correlated negatively with SUVmean (*r* = − 0.338). In the validation set, associations between factor 1 and TLG and MATV (*r* = 0.812, 0.887), factor 2 and SUVmax, SUVpeak and TLG (r = 0.838–0.876), and factor 3 and TLG and MATV (*r* = 0.494, 0.815, respectively) remained significant (Supplement 2). Low association was found between factors (Supplement 3).

**Table 2.** Correlations of radiomic factors with clinical parameters and FDG-PET parameters in the training set

	Factor 1	Factor 2	Factor 3	Factor 4	Factor 5	Factor 6	Factor 7	Factor 8	SUVmax	SUVpeak	SUVmean	TLG	MATV
**T-stage**	**0.454**	0.234	− 0.102	0.11	0.111	− 0.101	0.123	0.035	**0.412**	**0.438**	**0.422**	0.318	**0.405**
*p* value	0.000	0.017	0.306	0.269	0.263	0.312	0.214	0.725	0.000	0.000	0.000	0.001	0.000
**N-stage**	0.075	− 0.082	0.086	0.144	0.069	0.043	− 0.053	0.047	0.057	0.045	0.042	0.06	0.065
*p* value	0.452	0.408	0.389	0.148	0.488	0.670	0.596	0.639	0.568	0.650	0.677	0.544	0.515
**HPV**	− 0.259	− 0.273	0.126	0.006	− 0.149	0.055	0.009	0.036	− 0.329	− 0.332	− **0.338**	− 0.195	− 0.245
*p* value	0.008	0.005	0.205	0.951	0.132	0.584	0.925	0.722	0.001	0.001	0.000	0.048	0.012
**Smoking (PY)**	0.021	0.05	− 0.12	0.077	0.27	0.013	− 0.039	0.077	0.139	0.095	0.099	− 0.032	0.029
*p* value	0.835	0.613	0.227	0.437	0.006	0.894	0.699	0.442	0.160	0.340	0.319	0.751	0.769
**SUVmax**	**0.440**	**0.717**	− 0.093	**0.311**	0.08	0.044	0.165	0.168					
*p* value	0.000	0.000	0.353	0.001	0.425	0.660	0.096	0.089					
**SUVpeak**	**0.521**	**0.704**	− 0.034	0.284	0.042	0.02	0.146	0.177					
*p* value	0.000	0.000	0.731	0.004	0.675	0.838	0.141	0.073					
**SUVmean**	**0.468**	**0.740**	− 0.073	0.289	0.01	− 0.016	0.151	0.157					
*p* value	0.000	0.000	0.463	0.003	0.919	0.869	0.127	0.112					
**TLG**	**0.807**	0.172	**0.395**	0.079	0.04	0.01	0.07	− 0.114					
*p* value	0.000	0.082	0.000	0.429	0.686	0.920	0.482	0.254					
**MATV**	**0.947**	0.023	0.034	0.044	0.104	0.001	0.023	− 0.234					
*p* value	0.000	0.820	0.734	0.656	0.297	0.997	0.817	0.018					

Bold numbers were significantly correlated (*p* < 0.001), after the Bonferroni multiple testing correction

### Prognostic value of clinical, ^18^F-FDG-PET parameters, and radiomic factors in the training set

The significant predictors of **recurrence** were in the training set per clinical, PET parameter of radiomic factors separately; HPV-status; MATV; and factors 1 and 4 (Supplement 4).

The combination of clinical and ^18^F-FDG-PET parameters resulted in N-stage, HPV-status; and SUVmean as significant predictors (Supplement 5). The combination of clinical and radiomics parameters resulted in HPV-status; and factors 1, 4, 5 as significant predictors. The combination of clinical, ^18^F-FDG-PET, and radiomics parameters resulted in HPV-status, SUVmean, SUVpeak, factor 3, 4, and 6 as significant predictors (Supplement 4) and was significantly (*p* = 0.041; Supplement 5) most accurate to predict recurrences (CI = 0.796, SE = 0.045) as compared with other combinations (Table 3).

**Table 3:** Predictive accuracy of clinical parameters, PET-parameters, and radiomics factors separately and combined for the prediction of locoregional recurrence, metastasis, and death

	Recurrence prediction				Metastasis prediction				Overall survival prediction			
	Patients	Recurrences	Concordance index	SE	Patients	Distant metastasis	Concordance index	SE	Patients	Deaths	Concordance index	SE
**Clinical parameters** T-stage, N-stage, HPV-status, Smoking (PY)	103	27	0.699	0.049	103	10	0.690	0.097	103	37	0.691	0.043
**PET parameters** SUVmax, SUVmean, SUVpeak, TLG, MATV	103	27	0.616	0.065	103	10	0.759	0.062	103	37	0.711	0.041
**Radiomics parameters** Factor 1 to 8	103	27	0.716	0.055	103	10	0.746	0.079	103	37	0.714	0.05
**Combined clinical + PET parameters**	103	27	0.758	0.05	103	10	0.822	0.047	103	37	0.744	0.042
**Combined clinical + radiomics**	103	27	0.770	0.043	103	10	0.831	0.066	103	37	0.749	0.047
**Combined clinical + PET + radiomics**	103	27	0.796	0.045	103	10	0.945	0.029	103	37	0.750	0.046

The significant predictors for *distant metastasis* were in the training set per clinical, PET parameter of radiomic factors separately; only MATV (Supplement 3).

The combination of clinical and ^18^F-FDG-PET parameters resulted in N-stage and SUVmean as significant predictors (Supplement 4). The combination of clinical parameters, ^18^F-FDG-PET parameters, and radiomics resulted in only MATV as significant predictor (Supplement 4).

The significant predictors for **overall survival** were in the training set per clinical, PET parameter of radiomic factors separately; T-stage, HPV-status; MATV; factors 1 and 5 (Supplement 4).

The combination of clinical and ^18^F-FDG-PET parameters resulted in HPV-status and MATV as significant predictors (Supplement 4). The combination of clinical parameters and radiomics resulted in factors 1 and 5 as significant predictors.

The combination of clinical parameters, ^18^F-FDG-PET parameters, and radiomics resulted in HPV-status, SUVmax, SUVmean, factors 1 and 5 as significant predictors (Supplement 5) and was non-significantly (*p* > 0.05; Supplement 6) most predictive (CI = 0.750, SE = 0.046) as compared with other combinations (Table 3).

### Validation of the prognostic models

In the validation set, the prognostic accuracy of each trained model predicting the risk for recurrence, metastasis, and overall survival was validated (Table 4). This resulted in a validated CI = 0.645 (SE = 0.071) for recurrence, CI = 0.627 (SE = 0.094) for metastasis, and CI = 0.764 (SE = 0.062) for overall survival (Table 4 and Fig. 4).

**Table 4:** The accuracy of the prediction models for recurrence, metastasis, and overall survival in the training set and validated in the validation set. For the recurrence prediction, the combination of HPV, SUVmean, SUVpeak, factors 3, 4, and 6 was most accurate. For the metastasis prediction, the use of only MATV was most accurate. For overall survival prediction, the combination of HPV, SUVmax, SUVmean, factors 1 and 5 was most accurate

Final prediction models	Training set				Validation set			
	Patients	Events	Concordance index	SE	Patients	Events	Concordance index	SE
**Recurrence prediction model** HPV, SUVmean, SUVpeak, factor 3, factor 4, factor 6	103	27	0.779	0.050	71	19	0.645	0.071
**Metastasis prediction model** MATV	103	10	0.657	0.093	71	18	0.627	0.094
**Overall survival prediction model** HPV, SUVmax, SUVmean, factor 1, factor 5	103	37	0.751	0.045	71	22	0.764	0.052

Events: amount of recurrences in the recurrence prediction model; amount of distant metastases in the metastasis prediction model; amount of deaths in the overall survival prediction model

*SE* standard error

**Fig. 3:**
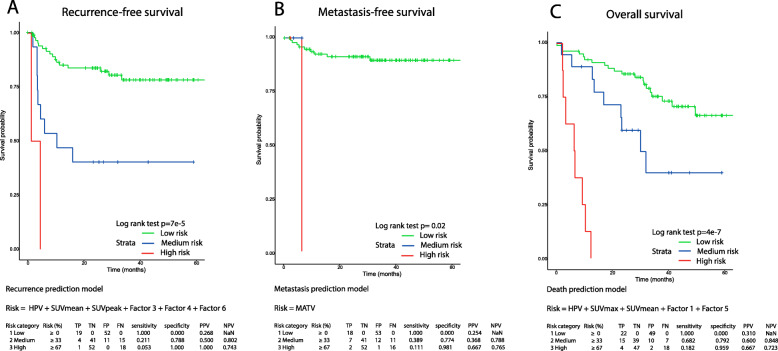
The accuracy of th e c ombined prediction of **a** locoregional recurrence, **b** metastasis, and **c** overall survival in the validation cohort. In **b**, the curve of the relatively small medium risk group for metastasis is short; this is due to the short follow-up time until the metastasis occurred. A significant predictive risk stratification (*p* < 0.05) was shown, divided in low (0–33%), medium (33–66%), and high (66–100%) risk for an unfavorable prognosis


The risk stratification into a high, medium, and low risk for adverse outcome was constructed; for recurrence (*p* = 7E−5), metastasis (*p* = 0.002) and overall survival (*p* = 4E−7) (Fig. 3, Supplement 7 and 8). A clinical applicable patient-specific risk calculator was constructed for a single patient to predict recurrence, metastasis, or death (Table 5).

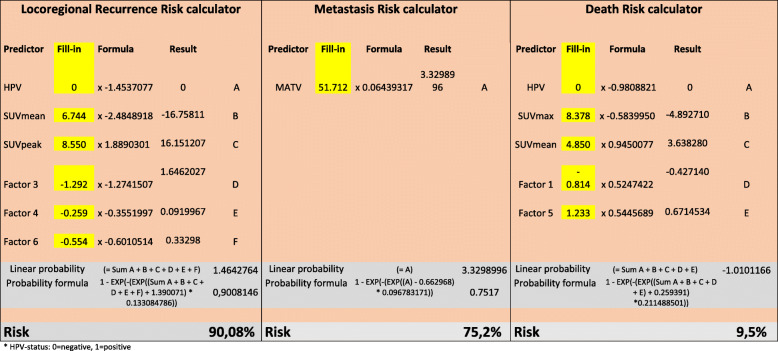

**Table 5:**
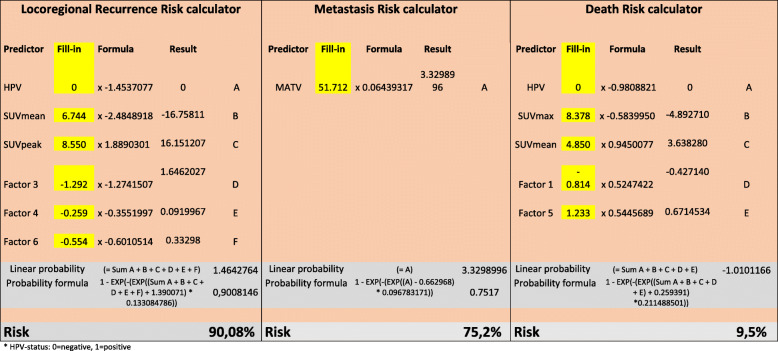
The risk for locoregional recurrence calculator, which can be used in clinical practice to calculate the risk per specific patient for locoregional recurrence during the follow-up time of 2 years. The yellow boxes could be filled-in with the single patient data in order to calculate the risk for locoregional recurrence. The risk for metastasis calculator, which can be used in clinical practice to calculate the risk per specific patient for metastasis during the follow-up time of 2 years. The yellow boxes are filled-in with the single patient data (with a large tumor) in order to calculate the risk for metastasis. The risk for death calculator, which can be used in clinical practice to calculate the risk per specific patient for death during the follow-up time of 2 years. The yellow boxes could be filled-in with the single patient data in order to calculate the risk for death

*HPV status: *0* = negative, *1* = positive

## Discussion

In this study, the examination of the prognostic value of pre-treatment ^18^F-FDG-PET radiomics in locally advanced HNSCC showed that the discriminatory performance of the combination of latent radiomics factors of ^18^F-FDG-PET was of additional value in predicting recurrence, metastasis, and overall survival and that the combination of clinical, PET, and radiomics parameters was most predictive.

### Radiomics process

The primary goal of radiomics is to build clinical models using machine learning techniques [44] in order to predict patient outcome, thereby allowing for better personalized treatment management. These multivariable prediction models might be unintelligible for clinicians, because they combine a large number of high-order multimodality image features [45, 46]. However, they may outperform visual analysis in terms of accuracy.

Aerts et al. [22] selected only the single best predictive features on CT from each of their four main feature categories (statistical features (e.g., mean, maximum, peak, mode), shape, grey-level-non-uniformity, and wavelet grey-level-non-uniformity HLH (i.e., describing intratumoral heterogeneity after decomposing the image in mid-frequencies). Bogowicz et al. [17] reported that performing PET, the combination of principle component analysis (PCA; a statistical procedure that converts a large set of observations of possibly correlated variables into a smaller projection of the most informative linearly uncorrelated variables) and univariate feature selection using the Cox regression with backward selection, resulted in the least complicated model with best discriminative power. However, their final PET model consisted of only 2 single radiomic features, and no clinical variables were considered. Vallières et al. [8] trained predictive models for each radiomic feature combined with clinical variables and patient outcome by performing random forests and made adjustments to model imbalance. Finally, only one PET-radiomics (GLN_GLSZM_) and two CT-radiomics features were included in the model. These methods manually excluded all other possible prognostic features.

In this study, a dimension reduction was performed of the feature space by removing redundant features (retaining 124 features). Based on these features, a factor analysis was performed, which consisted of a feature subset (i.e., factor) and contains a part of the predictive feature spectrum on a scale of importance. This allowed the preservation of the multiple predictive features and assess possible interactions or associations. This might provide insight in the underlying concepts of the heterogeneous whole-lesion PET data, as a basis for identification and targeting tumoral subvolumes which are predictive for adverse outcome [47]. Moreover, this factor analysis was done separately from the patient outcome, which might allow for the improvement of the tumor-specific classification, as basis for prognosis prediction. However, in other studies which selected single features, this inter-correlation of feature was lost [17, 22]. Thirdly, it overcomes the risk of data overfitting, which arises when the number of features is large and the number of training data is comparatively small [48].

### Tumor characteristics by radiomic factors

The spectrum of known predictive clinical and first-order PET parameters might be extended with non-correlated PET-radiomic features we found in this study, capturing complementary characteristics of the complex heterogeneous tumoral microenvironment.

Low values of factor 3, 4, and 6 were predictive of *recurrence*, complementary to negative HPV-status, low SUVmean, and high SUVpeak. Factor 3 correlated in the validation set with MATV and measured mainly maximum histogram gradient and long low grey-level lengths with a variance of lengths and zones, and high busyness, which might indicate tumoral intensity heterogeneity in tumoral zones of varying size, with long rows of low grey-level voxels (i.e., low FDG uptake). These features might capture the presence of necrotic regions within the core of tumors. Previously, this correlation between heterogeneity and volume in PET-data was reported by Hatt et al. [20]. Also Cheng et al. [49] found that besides TLG, uniformity (local scale texture parameter) and zone-size non-uniformity (ZSNU) were usable as prognostic stratifiers. This was confirmed by Vallières et al. [8], who also reported that GLSZM_GLN_ (grey-level size zone matrix with grey-level non-uniformity) was predictive for locoregional recurrence. Also Bogowicz et al. [17] found that GLSZM_ZSLGE_ (grey-level size zone matrix; with zone size low grey-level emphasis) was predictive for favorable prognosis (CI 0.71). However, in their study, different scanners were used between training and validation cohorts, which reduced data quality. Factor 4 measured slightly different characteristics such as intensity differences with high grey-level counts (inversed low grey-level count) and grey-level non-uniformity (inversed coarseness). This factor might capture the heterogeneity of tumoral sub-areas with a mainly high FDG-tracer uptake. Factor 6 measured the histogram variety of intensity and quantifies the complexity of the texture (second measure of information correlation), which might capture the tumoral range of FDG-uptake and differences of uptake between sub-areas. These radiomics features, bundled in factors, were not previously described in literature and might provide insights in the extent of tumoral clonal heterogeneity and interactions, which might help us to control tumors [6].

For *distant metastasis* prediction, we found in this study the use of MATV only was most accurate and outperformed all other clinical and radiomic parameters. This was partly confirmed by Vallières et al. [8], who also found tumoral volume, as well as age, tumor type, and N-stage as well as CT-radiomic heterogeneity features as predictive parameter. The large metabolic active tumor volume might enable large numbers of cell divisions, tumor progression into genetic instability, which might lead to metastatic ability [6].

High values of factors 1 and 5 were most predictive of adverse *overall survival*, complementary to negative HPV-status, SUVmax, and SUVmean. Factor 1 correlated significantly with T-stage and all PET parameters, with the highest correlation of those which were volume-related. This was in line with Vallieres et al. [8], who found that volume outperformed each radiomic models. However, factor 1 consisted also of mainly morphologic and non-uniformity texture features and was dependent on high intensity, which might correlate with large heterogeneous tumoral entities. This factor might capture the voluminous extent of the tumor, combined with areas of high FDG-tracer uptake. El Naqa et al. [23] also reported that intensity histogram and shape features were predictive of survival. Factor 5 measured also morphological tumor characteristics, such as asperity, major axis length, and inversed compactness and inversed flatness. This was found complementary to the volume-related features in factor 1, and in line with Bogowicz et al. [17], who reported that besides GLSZM_ZSLGE_, sphericity was most predictive for favorable prognosis (CI = 0.71). Also, Aerts et al. reported similar results in CT-data, showing that patients with more compact/spherical tumors had better survival probability [22]. Factors 1 and 2 both correlated with PET parameters and reflected particular heterogeneous distribution of FDG-PET uptake. Factor 1 correlated with volume-related TLG and MATV in the validation set. Factor 2 measured the histogram range, contrast, and small high grey emphasis, and correlated with SUVmax, SUVpeak, and SUVmean, and did not remain predictive.

### Discriminative power of prediction models

In order to improve predictive accuracy, patient-specific tumoral characteristics were captured by radiomics features and such as low grey-level zone sizes, heterogeneous busyness and morphologic tumor volume, and bundled by factors. Prediction models including these factors are hypothesized to be more patient-specific, because of more unique characteristics, than models which do not investigate underlying feature correlations and include only the single most predictive feature. Vallières et al. [8] combined clinical parameters, without HPV-status, with only one PET- and CT-radiomic feature; however, the prediction accuracy was similar for locoregional recurrences (AUC = 0.69) and overall survival (CI = 0.74). Aerts et al. [22] used the top 4 performing CT-features of each radiomics feature category, where inclusion of TNM-stage improved performance and showed a survival prediction of CI = 0.69. Bogowicz et al. [17] reported a CI of 0.71 using PET-radiomics; however, data was influenced by artifacts, scanner, and protocol heterogeneity. Also, current study showed that for metastasis prediction, the use of only MATV was most accurate. The accuracy of the prediction model combining all clinical (T-stage), first-order PET (SUVmean), and radiomic factors was found to be higher than the final model, consisting of only MATV. This might be due to the fact that the other features still hold some predictive power. Although this might provide insights in metastatic tumor characteristics, it should be validated in future studies. This was partly in line with Vallières et al. [8], who also found volume-parameter was most predictive, but they found additional value for CT-radiomics features.

### Clinical applicability

The efficacy of a treatment plan, nowadays based on information from clinical examination (under anesthesia), visual interpretation of imaging, and invasive biopsies, could be optimized by taking the patient-specific pathophysiologic phenotype into account [50] using quantitative imaging assessment. The underlying tumor biology could be heterogeneous with different sub-clonal populations, continuously changing and associated with resistance to treatment, recurrence, and overall survival [8, 22]. Many studies [8, 17, 22, 23] constructed predictive models based on the selection of a few radiomic features excluding clinical parameters (e.g., HPV status) and interactions with radiomic features, in order to reduce the risk for overfitting [8, 17, 22].

In this study, we showed an advanced factor analysis using three-dimensional whole-lesion radiomic features as well as retaining feature interactions captured in radiomic factors. These complementary factors improved predictive accuracy to the basis of clinical factors, including HPV-status and first-order PET parameters, and remained accurate after validation. Although we found a correlation between MATV and T-stage (mainly based on tumor volume), volume-related parameters were more predictive. Furthermore, we presented a patient-specific clinical-applicable risk stratification for patients with head and neck cancer treated with (chemo)radiotherapy. Low-risk patients could be candidates for treatment de-escalation studies [51, 52], whereas high-risk patients could benefit from treatment escalation [53], immunotherapy [54], or surgical treatment. This optimization of treatment efficacy might also result in a beneficial reduction of costs. Identification and validation of optimal machine-learning methods for radiomic applications using standardized EANM guidelines [26] is crucial towards reproducible biomarkers in clinical practice, complementary to the clinical and first-order PET parameters.

### Limitations

**Fig. 4:**
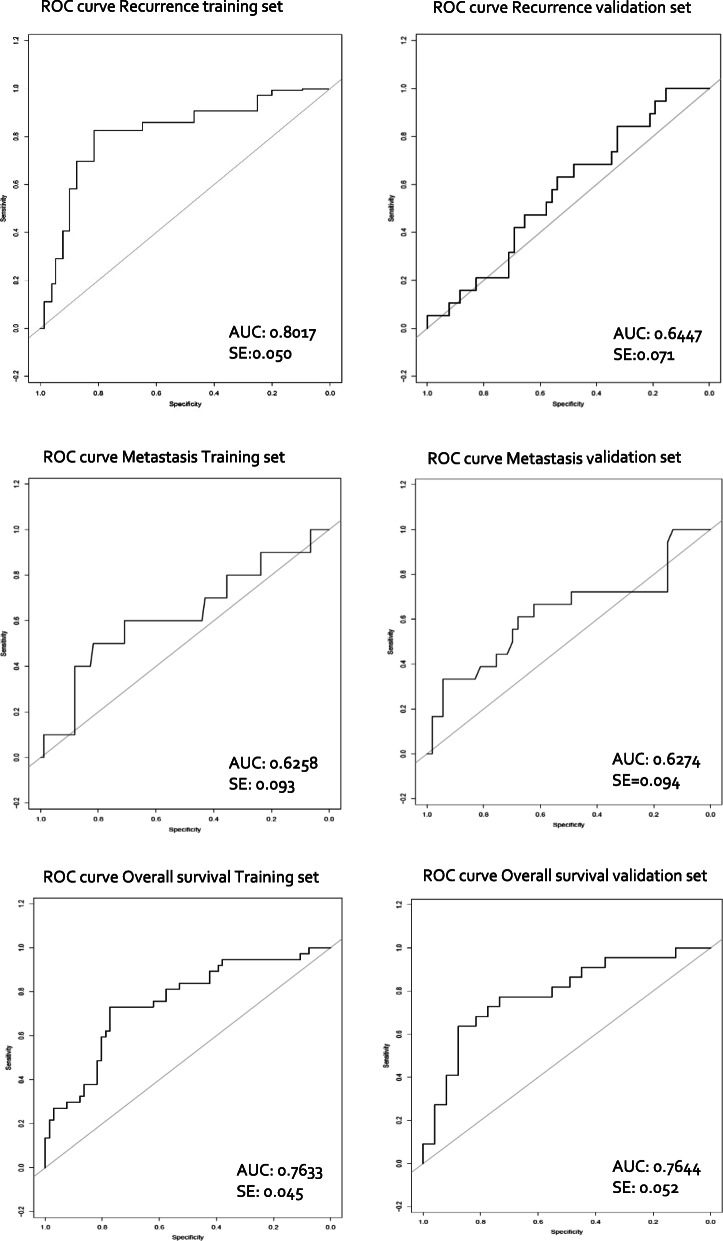
ROC curves in the training and validation set per patient outcome prediction. **A**rea under the incident receiver operating characteristic curve (ROC) for each final model in the training set as well as in the validation set for the prediction of recurrence, metastasis, and death within 2 years of follow-up after end of treatment

At the assessment of multiple clinical, first-order, and radiomic features, there is a risk for overfitting bias. In the current study, we used a relatively large patient sample size and performed a multicollinearity filtering to exclude highly correlated features. Moreover, the factor analysis projects the large and collinear radiomic feature-space onto an orthogonal latent-feature-space of smaller dimension (8 factors) while retaining the bulk of the information contained in the full data. This projection is thus geared towards the avoidance of overfitting. Finally, a limited amount of clinical, first-order PET and PET-radiomic factors was combined in a multivariable model. However, it is still possible that the number of events was not enough to construct a statistically robust prediction model. In this study, validation was performed internally by 5-fold cross-validation of the prognostic models. Moreover, we used an independent validation-cohort of similar institute to estimate the performance of a prediction model. In Table 4 and Fig. 4, we present the results obtained for the training set as well as the independent validation set. We can see that for the recurrence prediction model, the concordance index for the independent validation set is somewhat lower, while for the other 2 models, a similar performance was found between the training and (independent) validation dataset. However, in future studies, validation in a larger cohort from an external institute is still needed.

The prognostic model performance might be optimized by a stricter redundancy filtering to retain only complementary factors; however, in this study, we saved the inclusion of possible predictive underlying relationships of features. This model should be constructed using a limited amount of factors separate from patients outcome, in order to solely include predictive tumoral processes and to minimize cohort-dependent prognostic influences. Another improvement of the prognostic model performance might be the implementation of complementary predictive CT-radiomic features [22, 55, 56], which would require similar acquisition parameters, artifacts reduction techniques, and a larger patient population to overcome the risk of overfitting and should be evaluated in future studies.

This study was hypothesis generating and the feasibility was tested. However, in the next step to clinical translation, more extensive validation and refinement on larger and external datasets as well as evaluation of the clnical applicable calculators, is needed. Moreover, it is of interest to perform further technical validation, such as by the use of voxel randomization [57, 58]. Our study suggests that adding radiomics to the ^18^F-FDG-PET image analysis can improve prognostication as a step towards personalized treatment of HNSCC patients.

## Conclusion

The combination of HPV-status, first-order ^18^F-FDG-PET parameters, and complementary radiomic phenotype-specific factors improved time-to-event prediction most accurately. Predictive tumor-specific characteristics and interactions might be captured and retained using radiomic factors, which allows for personalized risk stratification and optimizing personalized cancer care.

## Supplementary information

**Additional file 1: **Supplement 1. All 8 radiomics factors, consisting of a spectrum of the extracted radiomics features. The number of each feature reflects the importance weight in that factor in which it is present. Supplement 2. Correlations of clinical parameters, 18F-FDG-PET-parameters and trained radiomic factors in the validation cohort. Supplement 3: The correlations between radiomics factors (Spearman’s Rho), with the significant correlated factors (bold) after Bonferroni’s correction (P< 0,00078125). Factor 1 was significantly correlated with factor 8. Factor 2 was significantly correlated with factor 7. Supplement 4. Multivariable cox regression analysis in the training set performing clinical, PET and/or radiomics parameters separately to predict recurrence, metastasis and overall survival. Multivariable cox regression analysis performing combined clinical, PET and/or radiomics parameters to predict recurrence, metastasis and overall survival. Supplement 6. The comparison of the predictive accuracy between the combined clinical + PET parameters and combined clinical + radiomics models versus the combination of clinical, + PET + radiomics predicting recurrence, distant metastasis and death. The prediction of recurrence was significantly more accurate using the combination of clinical + PET + radiomic factors than the combination of clinical + PET parameters, and it showed a borderline significant trend compared with clinical + radiomics factors. The prediction of metastasis was found significant more accurate combining clinical + PET + radiomics compared to clinical + PET and clinical+ radiomics factors. The prediction of overall survival was found not significant different for any prediction model. Supplement 7a. The risk stratification was constructed in the training set, using the combined prediction model for locoregional recurrence, metastasis and death (Figure 3). 7b. The risk stratification using the combined prediction model for locoregional recurrence, metastasis and death (Figure 3). 7c. The risk stratification using the combined prediction model for locoregional recurrence, metastasis and death (Figure 3). Supplement 8a. The risk stratification was validated in the validation set, using the combined prediction model for locoregional recurrence, metastasis and death (Figure 3). 8b. The risk stratification using the combined prediction model for locoregional recurrence, metastasis and death (Figure 3). 8c. The risk stratification using the combined prediction model for locoregional recurrence, metastasis and death (Figure 3). Supplement 9. TRIPOD Checklist: Prediction Model Development. Supplement 10. Output example of the RaCat tool

## Data Availability

The datasets used in this study are available from the corresponding author on reasonable request.

## Abbreviations

^18^F-FDG-PET: 18-Fluor-labeled Fluoro-2-deoxy-glucose positron emission tomography
AUC: Area under the curve
CI: Concordance index
CT: Computed tomography
HNSCC: Head and neck squamous cell carcinoma
HPV: Human papilloma virus
IBSI: International biomarker standardization initiative
LN: Lymph node metastasis
MATV: Metabolic active tumor volume
OS: Overall survival
PT: Primary tumor
SE: Standard error
SUV: Standard uptake volume
