# Supplementary material for: Predictive value of quantitative 18F-FDG-PET radiomics analysis in patients with head and neck squamous cell carcinoma
Source: EJNMMI Res. 2020 Sep 7;10:102. doi: 10.1186/s13550-020-00686-2 (PMC7477048; doi:10.1186/s13550-020-00686-2)
Supplement: Supplementary file 1 — Additional file 1: Supplement 1. All 8 radiomics factors, consisting of a spectrum of the extracted radiomics features. The number of each feature reflects the importance weight in that factor in which it is present. Supplement 2. Correlations of clinical parameters, 18F-FDG-PET-parameters and trained radiomic factors in the validation cohort. Supplement 3: The correlations between radiomics factors (Spearman’s Rho), with the significant correlated factors (bold) after Bonferroni’s correction (P< 0,00078125). Factor 1 was significantly correlated with factor 8. Factor 2 was significantly correlated with factor 7. Supplement 4. Multivariable cox regression analysis in the training set performing clinical, PET and/or radiomics parameters separately to predict recurrence, metastasis and overall survival. Multivariable cox regression analysis performing combined clinical, PET and/or radiomics parameters to predict recurrence, metastasis and overall survival. Supplement 6. The comparison of the predictive accuracy between the combined clinical + PET parameters and combined clinical + radiomics models versus the combination of clinical, + PET + radiomics predicting recurrence, distant metastasis and death. The prediction of recurrence was significantly more accurate using the combination of clinical + PET + radiomic factors than the combination of clinical + PET parameters, and it showed a borderline significant trend compared with clinical + radiomics factors. The prediction of metastasis was found significant more accurate combining clinical + PET + radiomics compared to clinical + PET and clinical+ radiomics factors. The prediction of overall survival was found not significant different for any prediction model. Supplement 7a. The risk stratification was constructed in the training set, using the combined prediction model for locoregional recurrence, metastasis and death (Figure 3). 7b. The risk stratification using the combined prediction model for locoregional recurr [file 13550_2020_686_MOESM1_ESM.docx]

**Supplements**

**Supplement 1.**

| **Radiomic FDG-PET Feature** | **Factor1** | **Factor2** | **Factor3** | **Factor4** | **Factor5** | **Factor6** | **Factor7** | **Factor8** |
| --- | --- | --- | --- | --- | --- | --- | --- | --- |
| Morphology_Surface_to_volume_ratio | -0,69 |  |  | -0,54 |  |  |  |  |
| **Morphology**_minor_axis_length | **0,84** |  |  |  |  |  |  |  |
| **Morphology**_least_axis_length | 0,9 |  |  |  |  |  |  |  |
| Intensity_histogram_Coefficient_of_variation | -0,57 |  |  |  |  | 0,51 |  |  |
| glcmFeatures2Dmrg_correlation | 0,67 |  |  | 0,47 |  |  |  |  |
| glcmFeatures2Dvmrg_inverse_difference_normalised | 0,77 |  |  |  |  |  |  | 0,33 |
| glcmFeatures2Dvmrg_inverse_difference_moment_normalised | 0,72 |  |  | 0,36 |  |  |  | 0,37 |
| glcmFeatures2Dvmrg_correlation | 0,68 |  |  | 0,3 |  | 0,45 |  | 0,32 |
| glcmFeatures3DWmrg_inverse_difference_normalised | 0,74 |  | 0,31 |  |  |  |  | 0,39 |
| glcmFeatures3DWmrg_inverse_difference_moment_normalised | 0,7 |  |  | 0,35 |  |  |  | 0,41 |
| glcmFeatures3DWmrg_correlation | 0,7 |  |  |  |  |  |  | 0,41 |
| glcmFeatures3DWmrg_second_measure_of_information_correlation | 0,6 | 0,37 |  | 0,36 |  | 0,37 |  |  |
| GLRLMFeatures2DWmrg_Grey_level_non_uniformity | 0,75 | -0,38 | 0,34 |  |  |  |  |  |
| **GLRLMFeatures2DWmrg**_**Run_length_non_uniformity** | **0,92** |  |  |  |  |  |  |  |
| GLRLMFeatures2Dvmrg_Grey_level_variance | 0,72 | 0,49 |  |  |  |  |  |  |
| **GLRLMFeatures3Dmrg**_**Run_length_non_uniformity** | **0,92** |  |  |  |  |  |  |  |
| GLSZMFeatures2Davg_Zone_size_non_uniformity | 0,77 | 0,43 |  |  |  |  |  |  |
| GLSZMFeatures3D_Large_zone_high_grey_level_emphasis | 0,68 |  |  |  |  |  |  | -0,41 |
| GLSZMFeatures3D_Zone_size_non_uniformity | 0,71 | 0,52 |  |  |  |  |  |  |
| **gldzmFeatures2Davg**_**Grey_level_non_uniformity**_GLDZM | 0,85 |  |  |  |  |  |  |  |
| **gldzmFeatures2Davg**_**Zone_distance_non_uniformity**_GLDZM | 0,81 |  |  |  |  |  |  |  |
| gldzmFeatures2Davg_Zone_distance_non_uniformity_normalized_GLDZM | -0,6 |  | 0,33 | -0,4 |  |  |  |  |
| **gldzmFeatures2Dmrg**_**Grey_level_non_uniformity**_GLDZM | **0,85** |  |  |  |  |  |  |  |
| **gldzmFeatures2Dmrg**_**Zone_distance_non_uniformity**_GLDZM | **0,85** |  |  |  | 0,33 |  |  |  |
| gldzmFeatures2Dmrg_Zone_distance_non_uniformity_normalized_GLDZM | -0,6 |  | 0,39 | -0,49 |  |  |  |  |
| **gldzmFeatures2Dmrg**_**Zone_distance_variance**_GLDZM | **0,86** |  |  |  |  |  |  |  |
| **gldzmFeatures3D**_**Zone_distance_non_uniformity**_GLDZM | **0,84** | 0,31 |  |  |  |  |  |  |
| gldzmFeatures3D_Zone_distance_variance_GLDZM | 0,58 | 0,47 |  |  |  |  |  |  |
| ngldmFeatures2Davg_Dependence_count_entropy | 0,7 | 0,32 |  | 0,47 |  |  |  |  |
| ngldmFeatures2Dmrg_High_dependence_high_grey_level_emphasis | 0,65 | 0,61 |  |  |  |  |  |  |
| ngldmFeatures2Dmrg_Dependence_count_entropy | 0,58 | 0,47 | -0,31 | 0,45 |  |  |  |  |
| ngldmFeatures3D_Low_dependence_low_grey_level_emphasis | -0,51 | -0,37 |  | -0,4 |  |  |  |  |
| **ngldmFeatures3D**_**High_dependence_high_grey_level_emphasis** | **0,81** | 0,39 |  |  |  |  |  |  |
| ngldmFeatures3D_Grey_level_non_uniformity | 0,71 |  | 0,47 |  |  |  |  |  |
| Statistics_minimum |  | 0,76 |  |  |  |  |  |  |
| **Intensity**_**histogram_range** | 0,35 | **0,8** |  |  |  |  |  |  |
| Intensity_histogram_Maximum_histogram_gradient_grey_level | 0,38 | 0,61 |  |  |  |  |  |  |
| Intensity_histogram_Minimum_histogram_gradient_grey_level | 0,44 | 0,67 |  |  |  |  |  |  |
| intensity_volume_int_at_vol_fraction_90 | 0,51 | 0,71 |  |  |  |  |  |  |
| **glcmFeatures2Davg**_**dissimilarity** |  | **0,85** |  |  |  |  |  |  |
| glcmFeatures2Davg_first_measure_of_information_correlation |  | -0,65 |  |  |  |  | -0,43 |  |
| glcmFeatures2Dmrg_inverse_variance |  | -0,77 |  |  |  |  | -0,32 |  |
| glcmFeatures2Dmrg_cluster_shade |  | -0,65 |  |  |  |  |  |  |
| glcmFeatures2Dmrg_first_measure_of_information_correlation |  | -0,7 |  | -0,49 |  |  |  |  |
| glcmFeatures3Davg_first_measure_of_information_correlation | 0,35 | -0,56 |  |  |  | -0,41 |  |  |
| glcmFeatures3Davg_second_measure_of_information_correlation |  | 0,58 | -0,31 | 0,42 |  | 0,37 |  |  |
| glcmFeatures3DWmrg_difference_entropy |  | 0,68 | -0,47 | 0,43 |  |  |  |  |
| **glcmFeatures3DWmrg_contrast** |  | **0,91** |  |  |  |  |  |  |
| glcmFeatures3DWmrg_inverse_variance |  | -0,72 |  | -0,37 |  |  | -0,42 |  |
| **glcmFeatures3DWmrg_cluster_prominence** |  | **0,81** |  |  |  |  | -0,31 |  |
| GLRLMFeatures3Dmrg_Run_entropy | 0,38 | 0,67 |  | 0,46 |  |  |  |  |
| GLSZMFeatures2Davg_Zone_size_non_uniformity_normalized |  | 0,71 | -0,36 |  |  |  | 0,47 |  |
| GLSZMFeatures3D_small_zone_emphasis |  | 0,62 | -0,36 | 0,47 |  |  |  |  |
| **GLSZMFeatures3D_Zone_size_non_uniformity_normalized** |  | **0,8** |  |  |  |  |  |  |
| **ngtdmFeatures2avg**_**complexity** |  | **0,91** |  |  |  |  |  |  |
| **ngtdmFeatures2avg**_**strength** |  | **0,88** |  |  |  |  |  |  |
| **ngtdmFeatures3D**_**contrast** |  | **0,82** |  |  |  |  |  |  |
| **ngtdmFeatures3D**_**strength** |  | **0,82** |  |  |  |  |  |  |
| **gldzmFeatures2Davg_Small_distance_high_grey_level_emphasis**_GLDZM |  | **0,9** |  |  |  |  |  |  |
| **gldzmFeatures2Dmrg**_Large_distance_high_grey_level_emphasis_GLDZM | 0,52 | 0,72 |  |  |  |  |  |  |
| **gldzmFeatures3D**_Large_distance_high_grey_level_emphasis_GLDZM | 0,31 | **0,86** |  |  |  |  |  |  |
| gldzmFeatures3D_Zone_distance_non_uniformity_normalized_GLDZM | -0,41 | -0,53 |  | -0,35 |  |  |  |  |
| ngldmFeatures2Dmrg_Low_dependence_emphasis |  | 0,58 | -0,53 | 0,43 |  |  |  |  |
| ngldmFeatures2Dmrg_Dependence_count_non_uniformity_normalized |  | 0,72 | -0,35 | 0,31 |  |  |  |  |
| ngldmFeatures3D_Low_dependence_emphasis |  | 0,78 | -0,33 | 0,31 |  |  |  |  |
| ngldmFeatures3D_Dependence_count_non_uniformity_normalized |  | 0,73 | -0,36 |  |  |  |  |  |
| Morphology_center_of_mass_shift |  | -0,31 | 0,68 |  |  |  |  |  |
| **Intensity**_**histogram_Maximum_histogram_gradient** |  |  | **0,81** |  |  |  |  |  |
| Intensity_histogram_Minimum_histogram_gradient | -0,41 |  | -0,79 |  |  |  |  |  |
| glcmFeatures2Dmrg_inverse_difference_moment |  | -0,53 | 0,56 | -0,51 |  |  |  |  |
| glcmFeatures2Dvmrg_first_measure_of_information_correlation |  |  | -0,67 |  |  | -0,48 |  |  |
| glcmFeatures3DWmrg_angular_second_moment |  |  | 0,72 | -0,55 |  |  |  |  |
| glcmFeatures3DWmrg_first_measure_of_information_correlation | -0,38 |  | -0,79 |  |  |  |  |  |
| **GLRLMFeatures2DWmrg**_**Long_run_low_grey_level_emphasis** |  |  | **0,86** | -0,37 |  |  |  |  |
| GLRLMFeatures2DWmrg_Run_percentage |  | 0,33 | -0,64 | 0,41 |  |  | 0,31 |  |
| GLRLMFeatures3Davg_Long_run_low_grey_level_emphasis |  |  | 0,79 | -0,31 |  |  |  |  |
| GLRLMFeatures3Dmrg_Run_length_non_uniformity_normalized |  | 0,38 | -0,76 | 0,41 |  |  |  |  |
| **GLRLMFeatures3Dmrg**_**Run_length_variance** |  |  | **0,91** |  |  |  |  |  |
| GLSZMFeatures2Davg_small_zone_emphasis |  | 0,46 | -0,57 | 0,51 |  |  |  |  |
| **GLSZMFeatures3D**_**Zone_size_variance** |  |  | **0,89** |  |  |  |  |  |
| **ngtdmFeatures2avg**_**busyness** |  |  | **0,83** |  |  |  |  |  |
| **ngtdmFeatures3D**_**busyness** |  |  | **0,93** |  |  |  |  |  |
| ngldmFeatures2Davg_Grey_level_non_uniformity | 0,53 | -0,32 | 0,69 |  |  |  |  |  |
| **ngldmFeatures2Dmrg**_**High_dependence_emphasis** |  |  | **0,86** | -0,32 |  |  |  |  |
| ngldmFeatures2Dmrg_Dependence_count_variance |  | -0,39 | 0,62 | -0,41 |  |  |  |  |
| **ngldmFeatures3D_Dependence_count_variance** |  |  | **0,85** |  |  |  |  |  |
| Morphology_Gearys_C |  |  | -0,33 | 0,58 |  |  |  | -0,37 |
| Statistics_Coefficient_of_variation |  | 0,33 |  | 0,58 |  | 0,37 |  |  |
| Statistics_Quartile_coefficient |  | 0,31 |  | 0,55 |  | 0,32 |  |  |
| intensity_volume_volume_at_int_fraction_90 |  |  | 0,34 | -0,54 |  |  |  |  |
| **intensity_volume_difference_vol_at_int_fraction** | 0,31 |  | -0,5 | 0,69 |  |  |  |  |
| **glcmFeatures2Davg**_second_measure_of_information_correlation |  |  | -0,35 | 0,69 |  |  |  |  |
| **glcmFeatures2Dmrg**_second_measure_of_information_correlation | 0,36 | 0,41 |  | 0,67 |  |  |  |  |
| **GLSZMFeatures2Dvmrg**_Small_zone_low_grey_level_emphasis | -0,32 | -0,35 |  | -0,68 |  |  | -0,31 |  |
| GLSZMFeatures3D_Zone_size_entropy | 0,44 | 0,36 | -0,47 | 0,55 |  |  |  |  |
| ngtdmFeatures2avg_coarseness | -0,48 | -0,4 |  | -0,55 |  |  |  |  |
| **ngtdmFeatures3D**_coarseness | -0,42 |  |  | -0,75 |  |  |  |  |
| **gldzmFeatures2Davg**_Grey_level_non_uniformity_normalized_GLDZM |  | -0,32 | 0,45 | -0,71 |  |  |  |  |
| **gldzmFeatures3D**_Large_distance_low_grey_level_emphasis_GLDZM |  |  | 0,39 | -0,71 |  |  |  |  |
| **gldzmFeatures3D**_Grey_level_non_uniformity_normalized_GLDZM |  | -0,33 | 0,57 | -0,61 |  |  |  |  |
| **ngldmFeatures2Davg**_Low_grey_level_count_emphasis |  |  | 0,57 | -0,69 |  |  |  |  |
| ngldmFeatures2Davg_Low_dependence_low_grey_level_emphasis | -0,42 | -0,4 |  | -0,58 |  |  |  |  |
| **ngldmFeatures2Davg**_dependence_Count_Energy | -0,41 |  |  | -0,71 |  |  |  |  |
| **ngldmFeatures2Dmrg**_dependence_Count_Energy | -0,41 | -0,33 | 0,39 | -0,63 |  |  |  |  |
| **ngldmFeatures3D**_dependence_Count_Energy | -0,51 | -0,36 |  | -0,63 |  |  |  |  |
| Morphology_Compactness2 | -0,39 |  |  |  | -0,77 |  |  |  |
| Morphology_asphericity | 0,33 |  |  |  | 0,79 |  |  |  |
| Morphology_major_axis_length | 0,58 |  |  |  | 0,65 |  |  |  |
| Morphology_elongation |  |  |  |  | -0,59 |  |  |  |
| Morphology_flatness |  |  |  |  | -0,75 |  |  |  |
| Intensity_histogram_kurtosis |  |  |  |  | 0,51 |  |  |  |
| glcmFeatures2Dvmrg_second_measure_of_information_correlation | 0,43 | 0,45 |  | 0,37 |  | 0,55 |  |  |
| GLSZMFeatures3D_Small_zone_low_grey_level_emphasis | -0,3 | -0,41 |  | -0,43 |  |  | -0,51 |  |
| Morphology_Morans_I | -0,34 |  |  | -0,45 |  | 0,49 |  |  |
| Intensity_histogram_skewness | -0,35 |  |  |  | 0,48 | 0,45 |  |  |
| Intensity_histogram_mode | 0,36 | 0,45 |  |  |  |  |  |  |
| Intensity_histogram_Quartile_coefficient | -0,46 |  |  |  |  |  |  |  |
| glcmFeatures3DWmrg_cluster_shade |  |  |  |  | 0,35 |  |  |  |
| ngtdmFeatures2avg_contrast |  | 0,46 |  |  |  |  |  |  |
| ngldmFeatures2Davg_Dependence_count_percentage | 0,3 |  |  |  |  |  | 0,47 |  |

**Supplement 1.** All 8 radiomics factors, consisting of a spectrum of the extracted radiomics features. The number of each feature reflects the importance weight in that factor in which it is present

|  | **Factor 1** | **Factor 2** | **Factor 3** | **Factor 4** | **Factor 5** | **Factor 6** | **Factor 7** | **Factor 8** | **SUVmax** | **SUVpeak** | **SUVmean** | **TLG** | **MATV** |
| --- | --- | --- | --- | --- | --- | --- | --- | --- | --- | --- | --- | --- | --- |
| **T-stage** | 0,361 | 0,177 | 0,032 | 0,263 | -0,109 | 0,061 | -0,034 | 0,053 | 0,359 | 0,37 | 0,347 | 0,354 | 0,21 |
| p-value | 0,002 | 0,140 | 0,792 | 0,027 | 0,365 | 0,612 | 0,778 | 0,661 | 0,002 | 0,002 | 0,003 | 0,002 | 0,079 |
| **N-stage** | 0,183 | 0,039 | 0,112 | -0,053 | 0,135 | -0,146 | -0,105 | 0,07 | 0,082 | 0,119 | 0,116 | 0,198 | 0,159 |
| p-value | 0,127 | 0,748 | 0,351 | 0,663 | 0,261 | 0,226 | 0,385 | 0,559 | 0,495 | 0,324 | 0,335 | 0,098 | 0,185 |
| **HPV** | -0,091 | -0,281 | -0,124 | 0,016 | -0,031 | 0,001 | 0,146 | 0,129 | -0,302 | -0,26 | -0,274 | -0,201 | -0,099 |
| p-value | 0,450 | 0,017 | 0,304 | 0,895 | 0,798 | 0,991 | 0,224 | 0,282 | 0,011 | 0,028 | 0,021 | 0,092 | 0,409 |
| **Alcohol (history)** | 0,037 | -0,085 | 0,17 | -0,235 | 0,016 | 0,064 | -0,218 | 0,108 | -0,082 | -0,098 | -0,086 | 0,012 | 0,02 |
| p-value | 0,761 | 0,480 | 0,157 | 0,049 | 0,894 | 0,595 | 0,068 | 0,371 | 0,495 | 0,416 | 0,476 | 0,924 | 0,866 |
| **Smoking (PY)** | 0,085 | -0,091 | 0,097 | -0,036 | 0,026 | 0,076 | -0,018 | 0,034 | -0,039 | -0,033 | -0,046 | 0,085 | 0,092 |
| p-value | 0,482 | 0,450 | 0,420 | 0,767 | 0,829 | 0,529 | 0,883 | 0,779 | 0,746 | 0,786 | 0,701 | 0,483 | 0,445 |
| **SUVmax** | 0,232 | **0,876** | -0,22 | 0,303 | -0,097 | -0,042 | -0,062 | 0,076 |  |  |  |  |  |
| p-value | 0,052 | 0,000 | 0,066 | 0,010 | 0,422 | 0,730 | 0,606 | 0,531 |  |  |  |  |  |
| **SUVpeak** | 0,318 | **0,838** | -0,21 | 0,308 | -0,144 | -0,073 | -0,098 | 0,151 |  |  |  |  |  |
| p-value | 0,007 | 0,000 | 0,079 | 0,009 | 0,230 | 0,546 | 0,418 | 0,208 |  |  |  |  |  |
| **SUVmean** | 0,259 | **0,864** | -0,239 | 0,267 | -0,173 | -0,102 | -0,074 | 0,103 |  |  |  |  |  |
| p-value | 0,029 | 0,000 | 0,044 | 0,024 | 0,149 | 0,400 | 0,540 | 0,394 |  |  |  |  |  |
| **TLG** | **0,887** | 0,246 | **0,494** | 0,379 | 0,178 | -0,181 | -0,196 | -0,184 |  |  |  |  |  |
| p-value | 0,000 | 0,038 | 0,000 | 0,001 | 0,138 | 0,132 | 0,101 | 0,124 |  |  |  |  |  |
| **MATV** | **0,812** | -0,106 | **0,815** | 0,399 | 0,225 | -0,06 | -0,248 | -0,386 |  |  |  |  |  |
| p-value | 0,000 | 0,381 | 0,000 | 0,001 | 0,060 | 0,616 | 0,037 | 0,001 |  |  |  |  |  |

**Supplement 2.**

**Supplement 2. Correlations of clinical parameters**, 18F-FDG-PET-parameters and trained radiomic factors in the **validation cohort**.

**Supplement 3.**

|  | **Factor 1** | **Factor 2** | **Factor 3** | **Factor 4** | **Factor 5** | **Factor 6** | **Factor 7** | **Factor 8** |
| --- | --- | --- | --- | --- | --- | --- | --- | --- |
| **Factor 1** | **-** | 0.045 | 0.278 | 0.098 | 0.026 | -0.078 | -0.124 | **0.501** |
| p-value |  | 0.653 | 0.005 | 0.327 | 0.794 | 0.434 | 0.212 | 0.000 |
| **Factor 2** |  | - | -0.022 | 0.026 | 0.017 | -0.066 | **-0.524** | 0.004 |
| p-value |  |  | 0,826 | 0,793 | 0,865 | 0,505 | 0 | 0,971 |
| **Factor 3** |  |  | - | 0.053 | 0.193 | 0.048 | -0.287 | 0.209 |
| p-value |  |  |  | 0.595 | 0.051 | 0.627 | 0.003 | 0.034 |
| **Factor 4** |  |  |  | - | -0,043 | -0,048 | 0,307 | -0,09 |
| p-value |  |  |  |  | 0,67 | 0,627 | 0,002 | 0,364 |
| **Factor 5** |  |  |  |  | - | -0,096 | 0,013 | -0,091 |
| p-value |  |  |  |  |  | 0,335 | 0,898 | 0,362 |
| **Factor 6** |  |  |  |  |  | - | -0,122 | 0,117 |
| p-value |  |  |  |  |  |  | 0,221 | 0,24 |
| **Factor 7** |  |  |  |  |  |  | - | -0,137 |
| p-value |  |  |  |  |  |  |  | 0,167 |
| **Factor 8** |  |  |  |  |  |  |  | - |
| p-value |  |  |  |  |  |  |  |  |

**Supplement 3**: The correlations between radiomics factors (Spearman’s Rho), with the significant correlated factors (bold) after Bonferroni’s correction (P< 0,00078125). Factor 1 was significantly correlated with factor 8. Factor 2 was significantly correlated with factor 7.

**Supplement 4. Multivariable event prediction in the training set.**

|  | **Recurrence prediction** | | | **Metastasis prediction** | | | **Overall survival prediction** | | |
| --- | --- | --- | --- | --- | --- | --- | --- | --- | --- |
| Number of pt 103 | P-value | HR | SE | P-value | HR | SE | P-value | HR | SE |
| Clinical parameters  T-stage  N-stage  HPV-status  Smoking (PY) | 0.870  0.504  0.002  0.728 | -  -  0.160  - | -  -  0.613  - | 0.235  0.505  0.693  0.736 | -  -  -  - | -  -  -  - | 0.054*  0.511  0.019*  0.610 | 1.232  -  1.109  - | 0.99-4.84  -  0.14-0.97  - |
| PET-parameters  SUVmax  SUVmean  SUVpeak  TLG  MATV | 0.907  0.526  0.623  0.975  0.005* | -  -  - -  1.043 | -  -  -  -  0.015 | 0.286  0.423  0.362  0.363  0.010* | -  -  -  -  1.062 | -  -  -  -  0.023 | 0.862  0.607  0.743  0.898  8.5e-7* | -  -  -  -  1.058 | -  -  -  -  0.011 |
| Radiomic factor  Factor 1  Factor 2  Factor 3  Factor 4  Factor 5  Factor 6  Factor 7  Factor 8 | 0.002*  0.937  0.458  0.027*  0.279  0.739  0.797  0.937 | 1.749  -  -  0.757  -  -  -  - | 0.180  -  -  0.126  -  -  -  - | 0.340  0.976  0.975  0.726  0602  0.799  0.504  0.884 | -  -  -  -  -  -  -  - | -  -  -  -  -  -  -  - | 2.1e-6*  0.565  0.767  0.391  8.1e-3*  0.620  0.834  0.790 | 1.960 -  -  -  1.582  -  -  - | 0.142  -  -  -  0.159  -  -  - |

* Parameters which remained significant after multivariable analysis and were included in the prediction models.

**Supplement 4.** Multivariable cox regression analysis in the **training set** performing clinical, PET and/or radiomics parameters separately to predict recurrence, metastasis and overall survival.

**Supplement 5. Multivariable event prediction of combined clinical, first-order PET and radiomic factors in the training set**

|  | **Recurrence prediction**  Events=27 | | | **Metastasis prediction**  Events=10 | | | **Overall survival prediction**  Events=37 | | |
| --- | --- | --- | --- | --- | --- | --- | --- | --- | --- |
| Number of pt 103 | P-value | HR | SE | P-value | HR | SE | P-value | HR | SE |
| Combined clinical + PET  T-stage  N-stage  HPV-status  Smoking  SUVmax  SUVmean  SUVpeak  TLG  MATV | 0.764  0.039  0.012  0.829  0.474  0.057  0.223  0.388  0.479 | -  0.398  0.156  -  -  0.249  -  -  - | -  0.446  0.735  -  -  0.732  -  -  - | 0.145  0.187  0.666  0.944  0.260  0.075  0.380  0.345  0.197 | 3.78  -  -  -  -  11.0  -  -  - | 0.913  -  -  -  -  1.348  -  -  - | 0.404  0.184  0.107  0.296  0.737  0.303  0.480  0.937  0.102 | -  -  0.443  -  -  -  -  -  1.055 | -  -  0.505  -  -  -  -  -  0.032 |
| Combined clinical + Radiomics  T-stage  N-stage  HPV-status  Smoking  Factor 1  Factor 2  Factor 3  Factor 4  Factor 5  Factor 6  Factor 7  Factor 8 | 0.517  0.063  0.017  0.485  0.005  0.839  0.113  0.069  0.048  0.276  0.781  0.569 | -  -  0.175  -  1.923  -  -  1.488  1.488  -  -  - | -  -  0.732  -  0.235  -  -  0.212  0.201  -  -  - | 0.181  0.390  0.408  0.201  0.539  0.382  0.904  0.562  0.132  0.304  0.131  0.802 | -  -  -  -  -  -  -  -  -  -  -  - | -  -  -  -  -  -  -  -  -  -  -  - | 0.763  0.169  0.175  0.500  0.0002  0.419  0.806  0.208  0.029  0.789  0.797  0.422 | -  -  -  -  1.917  -  -  -  1.416  -  -  - | -  -  -  -  0.172  -  -  -  0.165  -  -  - |
| Combined clinical + PET + radiomics  T-stage  N-stage  HPV-status  Smoking  SUVmax  SUVmean  SUVpeak  TLG  MATV  Factor 1  Factor 2  Factor 3  Factor 4  Factor 5  Factor 6  Factor 7  Factor 8 | 0.990  0.917  0.002*  0.932  0.971  0.0006*  0.0005*  0.876  0.979  0.965  0.979  0.042*  0.018*  0.993  0.003*  0.989  0.981 | -  -  0.140  -  -  0.034  13.18  -  -  -  -  0.192  0.782  -  0.445  -  - | -  -  0.646  -  -  0.990  0.7351  -  -  -  -  0.807  0.193  -  0.276  -  - | 0.611  0.555  0.552  0.623  0.542  0.628  0.610  0.597  0.020*  0.554  0.562  0.621  0.568  0.580  0.588  0.643  0.615 | -  -  -  -  -  -  -  -  4.250  -  -  -  -  -  -  -  - | -  -  -  -  -  -  -  -  0.621  -  -  -  -  -  -  -  - | 0.989  0.959  0.018*  0.995  0.024*  0.025*  0.984  0.983  0.998  0.0002*  0.996  0.994  0.917  0.0009*  0.994  0.998  0.971 | -  -  0.326  -  0.498  3.049  -  -  -  1.768  -  -  -  1.837  -  -  - | -  -  0.474  -  0.310  0.499  -  -  -  0.154  -  -  -  0.183  -  -  - |

* Parameters which remained significant after multivariable analysis and were included in the prediction models

**Supplement 5.** Multivariable cox regression analysis performing combined clinical, PET and/or radiomics parameters to predict recurrence, metastasis and overall survival.

**Supplement 6. The comparison of prediction models.**

|  | **Recurrence** | | **Metastasis** | | **Overall survival** | |
| --- | --- | --- | --- | --- | --- | --- |
|  | **Combined**  **clinical + PET-parameters**  **p-value** | **Combined**  **clinical + radiomics**  **p-value** | **Combined**  **clinical + PET-parameters**  **p-value** | **Combined**  **clinical + radiomics**  **p-value** | **Combined**  **clinical + PET-parameters**  **p-value** | **Combined clinical + radiomics**  **p-value** |
| **Combined clinical + radiomics** | 0.1619 | - | 0.653 | - | 0.1722 | - |
| **Combined clinical + PET+ radiomics** | **0.04146** | 0.05286 | **4.01 e-5** | **4.95 e-6** | 0.2988 | 0.4738 |

**Supplement 6.** The comparison of the predictive accuracy between the combined clinical + PET parameters and combined clinical + radiomics models versus the combination of clinical, + PET + radiomics predicting recurrence, distant metastasis and death. The prediction of recurrence was significantly more accurate using the combination of clinical + PET + radiomic factors than the combination of clinical + PET parameters, and it showed a borderline significant trend compared with clinical + radiomics factors. The prediction of metastasis was found significant more accurate combining clinical + PET + radiomics compared to clinical + PET and clinical+ radiomics factors. The prediction of overall survival was found not significant different for any prediction model.

**Supplement 7.**

| **A**  Recurrence prediction Training set | | | | | | | | |
| --- | --- | --- | --- | --- | --- | --- | --- | --- |
| Risk (%) | tp | tn | fp | fn | sens | spec | ppv | npv |
| 0 | 27 | 0 | 76 | 0 | 1.000 | 0.000 | 0.262 | NaN |
| 10 | 27 | 0 | 76 | 0 | 1.000 | 0.000 | 0.262 | NaN |
| 20 | 20 | 26 | 50 | 7 | 0.741 | 0.342 | 0.286 | 0.788 |
| 30 | 10 | 65 | 11 | 17 | 0.370 | 0.855 | 0.476 | 0.793 |
| 40 | 8 | 70 | 6 | 19 | 0.296 | 0.921 | 0.571 | 0.787 |
| 50 | 7 | 71 | 5 | 20 | 0.259 | 0.934 | 0.583 | 0.780 |
| 60 | 5 | 73 | 3 | 22 | 0.185 | 0.961 | 0.625 | 0.768 |
| 70 | 3 | 75 | 1 | 24 | 0.111 | 0.987 | 0.750 | 0.758 |
| 80 | 1 | 75 | 1 | 26 | 0.037 | 0.987 | 0.500 | 0.743 |
| 90 | 0 | 75 | 3 | 27 | 0.000 | 0.987 | 0.000 | 0.735 |

| **B** Metastasis prediction Training set | | | | | | | | |
| --- | --- | --- | --- | --- | --- | --- | --- | --- |
| Risk (%) | tp | tn | fp | fn | sensitivity | specificity | ppv | npv |
| 0 | 10 | 0 | 93 | 0 | 1.0 | 0.000 | 0.097 | NaN |
| 10 | 5 | 74 | 19 | 5 | 0.5 | 0.796 | 0.208 | 0.937 |
| 20 | 1 | 83 | 10 | 9 | 0.1 | 0.892 | 0.091 | 0.902 |
| 30 | 1 | 89 | 4 | 9 | 0.1 | 0.957 | 0.200 | 0.908 |
| 40 | 1 | 91 | 2 | 9 | 0.1 | 0.978 | 0.333 | 0.910 |
| 50 | 1 | 92 | 1 | 9 | 0.1 | 0.989 | 0.500 | 0.911 |
| 60 | 1 | 92 | 1 | 9 | 0.1 | 0.989 | 0.500 | 0.911 |
| 70 | 1 | 92 | 1 | 9 | 0.1 | 0.989 | 0.500 | 0.911 |
| 80 | 0 | 92 | 1 | 10 | 0.0 | 0.989 | 0 | 0.902 |

| **C** Death prediction Training set | | | | | | | | |
| --- | --- | --- | --- | --- | --- | --- | --- | --- |
| Risk (%) | tp | tn | fp | fn | sensitivity | specificity | ppv | npv |
| 0 | 37 | 0 | 66 | 0 | 1.000 | 0.000 | 0.359 | NaN |
| 10 | 34 | 25 | 41 | 3 | 0.919 | 0.379 | 0.453 | 0.893 |
| 20 | 28 | 41 | 25 | 9 | 0.757 | 0.621 | 0.528 | 0.820 |
| 30 | 18 | 53 | 13 | 19 | 0.486 | 0.803 | 0.581 | 0.736 |
| 40 | 11 | 61 | 5 | 26 | 0.297 | 0.924 | 0.688 | 0.701 |
| 50 | 10 | 64 | 2 | 27 | 0.270 | 0.970 | 0.833 | 0.703 |
| 60 | 8 | 64 | 2 | 29 | 0.216 | 0.970 | 0.800 | 0.688 |
| 70 | 5 | 65 | 1 | 32 | 0.135 | 0.985 | 0.833 | 0.670 |
| 80 | 4 | 66 | 0 | 33 | 0.108 | 1.000 | 1.000 | 0.667 |

**Supplement 7a.** The risk stratification was constructed in the training set, using the combined prediction model for locoregional recurrence, metastasis and death (Figure 3). **7b.** The risk stratification using the combined prediction model for locoregional recurrence, metastasis and death (Figure 3). 7**c.** The risk stratification using the combined prediction model for locoregional recurrence, metastasis and death (Figure 3).

**Supplement 8.**

| **A**  Recurrence prediction Validation set | | | | | | | | |
| --- | --- | --- | --- | --- | --- | --- | --- | --- |
| Risk (%) | tp | tn | fp | fn | sens | spec | ppv | npv |
| 0 | 19 | 0 | 52 | 0 | 1.000 | 0.000 | 0.268 | NaN |
| 10 | 19 | 0 | 52 | 0 | 1.000 | 0.000 | 0.268 | NaN |
| 20 | 16 | 16 | 36 | 3 | 0.842 | 0.308 | 0.308 | 0.842 |
| 30 | 4 | 41 | 11 | 15 | 0.211 | 0.788 | 0.267 | 0.732 |
| 40 | 2 | 47 | 5 | 17 | 0.105 | 0.904 | 0.286 | 0.734 |
| 50 | 1 | 51 | 1 | 18 | 0.053 | 0.981 | 0.500 | 0.739 |
| 60 | 1 | 52 | 0 | 18 | 0.053 | 1.000 | 1.000 | 0.743 |
| 70 | 1 | 52 | 0 | 18 | 0.053 | 1.000 | 1.000 | 0.743 |
| 80 | 1 | 52 | 0 | 18 | 0.053 | 1.000 | 1.000 | 0.743 |
| 90 | 1 | 52 | 0 | 18 | 0.053 | 1.000 | 1.000 | 0.743 |

| **B** Metastasis prediction Validation set | | | | | | | | |
| --- | --- | --- | --- | --- | --- | --- | --- | --- |
| Risk (%) | tp | tn | fp | fn | sensitivity | specificity | ppv | npv |
| 0 | 18 | 0 | 53 | 0 | 1.000 | 0.000 | 0.254 | NaN |
| 10 | 18 | 0 | 53 | 0 | 1.000 | 0.000 | 0.254 | NaN |
| 20 | 13 | 18 | 35 | 5 | 0.722 | 0.340 | 0.271 | 0.783 |
| 30 | 7 | 41 | 12 | 11 | 0.389 | 0.774 | 0.368 | 0.788 |
| 40 | 5 | 50 | 3 | 13 | 0.278 | 0.943 | 0.625 | 0.794 |
| 50 | 4 | 50 | 3 | 14 | 0.222 | 0.943 | 0.571 | 0.781 |
| 60 | 2 | 52 | 1 | 16 | 0.111 | 0.981 | 0.667 | 0.765 |
| 70 | 1 | 52 | 1 | 17 | 0.056 | 0.981 | 0.500 | 0.754 |
| 80 | 1 | 52 | 1 | 17 | 0.056 | 0.981 | 0.500 | 0.754 |

| **C** Death prediction Validation set | | | | | | | | |
| --- | --- | --- | --- | --- | --- | --- | --- | --- |
| Risk (%) | tp | tn | fp | fn | sensitivity | specificity | ppv | npv |
| 0 | 22 | 0 | 49 | 0 | 1.000 | 0.000 | 0.310 | NaN |
| 10 | 21 | 11 | 38 | 1 | 0.955 | 0.224 | 0.356 | 0.917 |
| 20 | 17 | 27 | 22 | 5 | 0.773 | 0.551 | 0.436 | 0.844 |
| 30 | 15 | 39 | 10 | 7 | 0.682 | 0.792 | 0.600 | 0.848 |
| 40 | 9 | 43 | 6 | 13 | 0.409 | 0.878 | 0.600 | 0.768 |
| 50 | 6 | 47 | 2 | 16 | 0.273 | 0.959 | 0.750 | 0.746 |
| 60 | 4 | 47 | 2 | 18 | 0.182 | 0.959 | 0.667 | 0.723 |
| 70 | 2 | 47 | 2 | 20 | 0.091 | 0.959 | 0.500 | 0.701 |
| 80 | 2 | 48 | 1 | 20 | 0.091 | 0.980 | 0.667 | 0.706 |
| 90 | 1 | 49 | 0 | 21 | 0.045 | 1.000 | 1.000 | 0.700 |

**Supplement 8a.** The risk stratification was validated in the validation set, using the combined prediction model for locoregional recurrence, metastasis and death (Figure 3). **8b.** The risk stratification using the combined prediction model for locoregional recurrence, metastasis and death (Figure 3). 8**c.** The risk stratification using the combined prediction model for locoregional recurrence, metastasis and death (Figure 3).

**Supplement 9.**


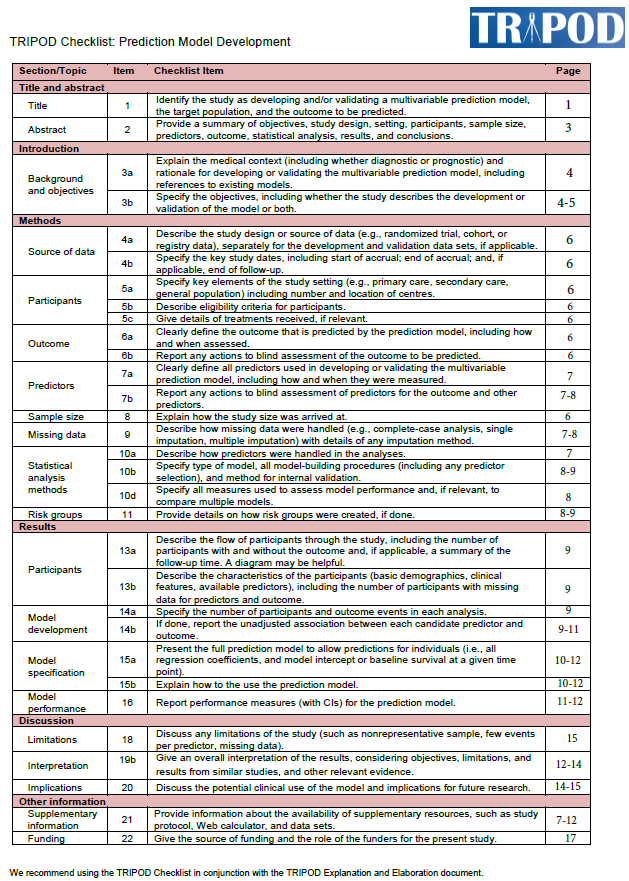


**Supplement 10. Output example of the RaCat tool**

| Patient Details | Patient Name | Patient000000 |
| --- | --- | --- |
| Patient Details | PatientID | 0 |
| Patient Details | Scan start | 0:00:00 |
| Patient Details | Scan Date | 20200708 |
| Dispersity | NumberLesions | 1 |
| Dispersity | DmaxPatient | 0 |
| Dispersity | DmaxBulk | 0 |
| Dispersity | SpreadBulk | 0 |
| Dispersity | SpreadPatient | 0 |
| Dispersity | DvolPatient | 0 |
| Dispersity | VolSpreadBulk | 0 |
| Dispersity | VolSpreadPatient | 0 |
| Dispersity | DSUVmaxBulk | 0 |
| Dispersity | DSUVmaxPatient | 0 |
| Dispersity | DSUVmaxSumBulk | 0 |
| Dispersity | DSUVmaxSumPatient | 0 |
| Dispersity | DSUVmaxSumHot | 0 |
| Dispersity | DSUVpeakBulk | 0 |
| Dispersity | DSUVpeakPatient | 0 |
| Dispersity | DSUVpeakSumBulk | 0 |
| Dispersity | DSUVpeakSumPatient | 0 |
| Dispersity | DSUVpeakSumHot | 0 |
| Morphology | Volume | 17865.6 |
| Morphology | approximate volume | 23696 |
| Morphology | Surface | 3802.99 |
| Morphology | Surface to volume ratio | 0.212866 |
| Morphology | Compactness1 | 0.0429789 |
| Morphology | Compactness2 | 0.656317 |
| Morphology | Spherical disproportion | 11.507 |
| Morphology | sphericity | 0.869036 |
| Morphology | asphericity | 0 |
| Morphology | center of mass shift | 22.377 |
| Morphology | maximum 3D diameter | 385.227 |
| Morphology | major axis length | 324.814 |
| Morphology | minor axis length | 319.251 |
| Morphology | least axis length | 317.325 |
| Morphology | elongation | 0.991399 |
| Morphology | flatness | 0.988405 |
| Morphology | vol density AABB | 0.382922 |
| Morphology | area density AABB | 0.489067 |
| Morphology | vol density AEE | 103.693 |
| Morphology | integrated intensity | 137455 |
| Morphology | Morans I | 0.0687269 |
| Morphology | Gearys C | 0.862496 |
| Local intensity | local intensity peak | 837.012 |
| Local intensity | global intensity peak | 902.432 |
| Statistics | mean | 769.383 |
| Statistics | variance | 204.697 |
| Statistics | skewness | -0.677025 |
| Statistics | kurtosis | -0.684809 |
| Statistics | median | 814.511 |
| Statistics | minimum | 387.049 |
| Statistics | 10th percentile | 552.756 |
| Statistics | 90th percentile | 916.263 |
| Statistics | maximum | 971.795 |
| Statistics | Interquartile range | 23.043 |
| Statistics | range | 584.746 |
| Statistics | Mean absolut deviation | 123.184 |
| Statistics | Robust mean absolute deviation | 125.082 |
| Statistics | Median absolute deviation | 119.768 |
| Statistics | Coefficient of variation | 0.185957 |
| Statistics | Quartile coefficient | 0.14889 |
| Statistics | Energy | 181399 |
| Statistics | Root mean | 782.573 |
| intensity volume | volume at int fraction 10 | 0.951047 |
| intensity volume | volume at int fraction 90 | 0 |
| intensity volume | int at vol fraction 10 | 10.85 |
| intensity volume | int at vol fraction 90 | 6.85 |
| intensity volume | difference vol at int fraction | 0.951047 |
| intensity volume | difference int at volume fraction | 4 |
| Intensity histogram | mean | 312.822 |
| Intensity histogram | variance | 328.495 |
| Intensity histogram | skewness | -0.672355 |
| Intensity histogram | kurtosis | -0.692202 |
| Intensity histogram | median | 33 |
| Intensity histogram | minimum | 16 |
| Intensity histogram | 10th percentile | 23 |
| Intensity histogram | 90th percentile | 37 |
| Intensity histogram | maximum | 39 |
| Intensity histogram | mode | 17 |
| Intensity histogram | Interquartile range | 9 |
| Intensity histogram | range | 23 |
| Intensity histogram | Mean absolut deviation | 493.737 |
| Intensity histogram | Robust mean absolute deviation | 412.663 |
| Intensity histogram | Median absolut deviation | 479.136 |
| Intensity histogram | Coefficient of variation | 0.183217 |
| Intensity histogram | Quartile coefficient | 0.142857 |
| Intensity histogram | Entropy | 420.483 |
| Intensity histogram | Uniformity | 0.0671736 |
| Intensity histogram | Energy | 3,00E+11 |
| Intensity histogram | Maximum histogram gradient | 146 |
| Intensity histogram | Maximum histogram gradient grey level | 35 |
| Intensity histogram | Minimum histogram gradient | -131 |
| Intensity histogram | Minimum histogram gradient grey level | 37 |
| glcmFeatures2Davg | joint maximum | 0.113339 |
| glcmFeatures2Davg | joint average | 302.707 |
| glcmFeatures2Davg | joint variance | 189.747 |
| glcmFeatures2Davg | joint entropy | 575.451 |
| glcmFeatures2Davg | difference average | 215.665 |
| glcmFeatures2Davg | difference variance | 395.315 |
| glcmFeatures2Davg | difference entropy | 243.651 |
| glcmFeatures2Davg | sum average | 605.415 |
| glcmFeatures2Davg | sum variance | 668.723 |
| glcmFeatures2Davg | sum entropy | 419.718 |
| glcmFeatures2Davg | angular second moment | 0.0623167 |
| glcmFeatures2Davg | contrast | 902.647 |
| glcmFeatures2Davg | dissimilarity | 215.665 |
| glcmFeatures2Davg | inverse difference | 0.478524 |
| glcmFeatures2Davg | inverse difference normalised | 0.949923 |
| glcmFeatures2Davg | inverse difference moment | 0.415249 |
| glcmFeatures2Davg | inverse difference moment normalised | 0.994195 |
| glcmFeatures2Davg | inverse variance | 0.366527 |
| glcmFeatures2Davg | correlation | 0.673736 |
| glcmFeatures2Davg | autocorrelation | 950.238 |
| glcmFeatures2Davg | cluster tendency | 668.724 |
| glcmFeatures2Davg | cluster shade | -455.501 |
| glcmFeatures2Davg | cluster prominence | 14862.3 |
| glcmFeatures2Davg | first measure of information correlation | -0.397596 |
| glcmFeatures2Davg | second measure of information correlation | 0.953978 |
| glcmFeatures2DDmrg | joint maximum | 0.0689661 |
| glcmFeatures2DDmrg | joint average | 321.792 |
| glcmFeatures2DDmrg | joint variance | 273.196 |
| glcmFeatures2DDmrg | joint entropy | 718.797 |
| glcmFeatures2DDmrg | difference average | 232.934 |
| glcmFeatures2DDmrg | difference variance | 478.217 |
| glcmFeatures2DDmrg | difference entropy | 281.767 |
| glcmFeatures2DDmrg | sum average | 643.585 |
| glcmFeatures2DDmrg | sum variance | 989.754 |
| glcmFeatures2DDmrg | sum entropy | 49.848 |
| glcmFeatures2DDmrg | angular second moment | 0.0152727 |
| glcmFeatures2DDmrg | contrast | 103.032 |
| glcmFeatures2DDmrg | dissimilarity | 232.934 |
| glcmFeatures2DDmrg | inverse difference | 0.463972 |
| glcmFeatures2DDmrg | inverse difference normalised | 0.946204 |
| glcmFeatures2DDmrg | inverse difference moment | 0.395886 |
| glcmFeatures2DDmrg | inverse difference moment normalised | 0.993385 |
| glcmFeatures2DDmrg | inverse variance | 0.334333 |
| glcmFeatures2DDmrg | correlation | 0.81033 |
| glcmFeatures2DDmrg | autocorrelation | 1057.69 |
| glcmFeatures2DDmrg | cluster tendency | 989.753 |
| glcmFeatures2DDmrg | cluster shade | -796.995 |
| glcmFeatures2DDmrg | cluster prominence | 25865.9 |
| glcmFeatures2DDmrg | first measure of information correlation | -0.229803 |
| glcmFeatures2DDmrg | second measure of information correlation | 0.912719 |
| glcmFeatures2Dmrg | joint maximum | 0.102149 |
| glcmFeatures2Dmrg | joint average | 302.682 |
| glcmFeatures2Dmrg | joint variance | 190.075 |
| glcmFeatures2Dmrg | joint entropy | 623.891 |
| glcmFeatures2Dmrg | difference average | 214.642 |
| glcmFeatures2Dmrg | difference variance | 404.748 |
| glcmFeatures2Dmrg | difference entropy | 258.068 |
| glcmFeatures2Dmrg | sum average | 605.363 |
| glcmFeatures2Dmrg | sum variance | 670.586 |
| glcmFeatures2Dmrg | sum entropy | 439.039 |
| glcmFeatures2Dmrg | angular second moment | 0.045213 |
| glcmFeatures2Dmrg | contrast | 897.136 |
| glcmFeatures2Dmrg | dissimilarity | 214.642 |
| glcmFeatures2Dmrg | inverse difference | 0.481276 |
| glcmFeatures2Dmrg | inverse difference normalised | 0.950155 |
| glcmFeatures2Dmrg | inverse difference moment | 0.418058 |
| glcmFeatures2Dmrg | inverse difference moment normalised | 0.99423 |
| glcmFeatures2Dmrg | inverse variance | 0.362226 |
| glcmFeatures2Dmrg | correlation | 0.688781 |
| glcmFeatures2Dmrg | autocorrelation | 950.111 |
| glcmFeatures2Dmrg | cluster tendency | 670.586 |
| glcmFeatures2Dmrg | cluster shade | -461.512 |
| glcmFeatures2Dmrg | cluster prominence | 15017.9 |
| glcmFeatures2Dmrg | first measure of information correlation | -0.22512 |
| glcmFeatures2Dmrg | second measure of information correlation | 0.86938 |
| glcmFeatures2Dvmrg | joint maximum | 0.0690467 |
| glcmFeatures2Dvmrg | joint average | 321.763 |
| glcmFeatures2Dvmrg | joint variance | 273.495 |
| glcmFeatures2Dvmrg | joint entropy | 727.574 |
| glcmFeatures2Dvmrg | difference average | 232.263 |
| glcmFeatures2Dvmrg | difference variance | 484.665 |
| glcmFeatures2Dvmrg | difference entropy | 285.317 |
| glcmFeatures2Dvmrg | sum average | 643.526 |
| glcmFeatures2Dvmrg | sum variance | 991.569 |
| glcmFeatures2Dvmrg | sum entropy | 499.624 |
| glcmFeatures2Dvmrg | angular second moment | 0.0150456 |
| glcmFeatures2Dvmrg | contrast | 102.412 |
| glcmFeatures2Dvmrg | dissimilarity | 232.263 |
| glcmFeatures2Dvmrg | inverse difference | 0.464467 |
| glcmFeatures2Dvmrg | inverse difference normalised | 0.946343 |
| glcmFeatures2Dvmrg | inverse difference moment | 0.396438 |
| glcmFeatures2Dvmrg | inverse difference moment normalised | 0.993424 |
| glcmFeatures2Dvmrg | inverse variance | 0.334707 |
| glcmFeatures2Dvmrg | correlation | 0.812771 |
| glcmFeatures2Dvmrg | autocorrelation | 1057.54 |
| glcmFeatures2Dvmrg | cluster tendency | 991.568 |
| glcmFeatures2Dvmrg | cluster shade | -801.688 |
| glcmFeatures2Dvmrg | cluster prominence | 25982.5 |
| glcmFeatures2Dvmrg | first measure of information correlation | -0.208993 |
| glcmFeatures2Dvmrg | second measure of information correlation | 0.903855 |
| glcmFeatures3Davg | joint maximum | 0.0643303 |
| glcmFeatures3Davg | joint average | 323.755 |
| glcmFeatures3Davg | joint variance | 261.463 |
| glcmFeatures3Davg | joint entropy | 725.079 |
| glcmFeatures3Davg | difference average | 259.685 |
| glcmFeatures3Davg | difference variance | 624.459 |
| glcmFeatures3Davg | difference entropy | 295.235 |
| glcmFeatures3Davg | sum average | 64.751 |
| glcmFeatures3Davg | sum variance | 914.522 |
| glcmFeatures3Davg | sum entropy | 493.282 |
| glcmFeatures3Davg | angular second moment | 0.0147495 |
| glcmFeatures3Davg | contrast | 131.331 |
| glcmFeatures3Davg | dissimilarity | 259.685 |
| glcmFeatures3Davg | inverse difference | 0.445669 |
| glcmFeatures3Davg | inverse difference normalised | 0.940829 |
| glcmFeatures3Davg | inverse difference moment | 0.376826 |
| glcmFeatures3Davg | inverse difference moment normalised | 0.991636 |
| glcmFeatures3Davg | inverse variance | 0.330772 |
| glcmFeatures3Davg | correlation | 0.744242 |
| glcmFeatures3Davg | autocorrelation | 1067.81 |
| glcmFeatures3Davg | cluster tendency | 914.522 |
| glcmFeatures3Davg | cluster shade | -714.743 |
| glcmFeatures3Davg | cluster prominence | 22333.7 |
| glcmFeatures3Davg | first measure of information correlation | -0.196152 |
| glcmFeatures3Davg | second measure of information correlation | 0.877805 |
| glcmFeatures3DWmrg | joint maximum | 0.0645152 |
| glcmFeatures3DWmrg | joint average | 32.367 |
| glcmFeatures3DWmrg | joint variance | 262.533 |
| glcmFeatures3DWmrg | joint entropy | 737.537 |
| glcmFeatures3DWmrg | difference average | 258.368 |
| glcmFeatures3DWmrg | difference variance | 632.167 |
| glcmFeatures3DWmrg | difference entropy | 29.932 |
| glcmFeatures3DWmrg | sum average | 647.339 |
| glcmFeatures3DWmrg | sum variance | 920.162 |
| glcmFeatures3DWmrg | sum entropy | 495.202 |
| glcmFeatures3DWmrg | angular second moment | 0.0143489 |
| glcmFeatures3DWmrg | contrast | 129.971 |
| glcmFeatures3DWmrg | dissimilarity | 258.368 |
| glcmFeatures3DWmrg | inverse difference | 0.446601 |
| glcmFeatures3DWmrg | inverse difference normalised | 0.941095 |
| glcmFeatures3DWmrg | inverse difference moment | 0.377816 |
| glcmFeatures3DWmrg | inverse difference moment normalised | 0.99172 |
| glcmFeatures3DWmrg | inverse variance | 0.331047 |
| glcmFeatures3DWmrg | correlation | 0.752468 |
| glcmFeatures3DWmrg | autocorrelation | 1067.37 |
| glcmFeatures3DWmrg | cluster tendency | 920.162 |
| glcmFeatures3DWmrg | cluster shade | -731.428 |
| glcmFeatures3DWmrg | cluster prominence | 22776.4 |
| glcmFeatures3DWmrg | first measure of information correlation | -0.167548 |
| glcmFeatures3DWmrg | second measure of information correlation | 0.86048 |
| GLRLMFeatures2Davg | short run emphasis | 0.872225 |
| GLRLMFeatures2Davg | long runs emphasis | 191.017 |
| GLRLMFeatures2Davg | Low grey level run emphasis | 0.00141195 |
| GLRLMFeatures2Davg | High grey level run emphasis | 876.041 |
| GLRLMFeatures2Davg | Short run low grey level emphasis | 0.00125684 |
| GLRLMFeatures2Davg | Short run high grey level emphasis | 743.223 |
| GLRLMFeatures2Davg | Long run low grey level emphasis | 0.00236174 |
| GLRLMFeatures2Davg | Long run high grey level emphasis | 1910.88 |
| GLRLMFeatures2Davg | Grey level non uniformity | 960.112 |
| GLRLMFeatures2Davg | Grey level non uniformity normalized | 0.10921 |
| GLRLMFeatures2Davg | Run length non uniformity | 99.397 |
| GLRLMFeatures2Davg | Run length non uniformity normalized | 0.767367 |
| GLRLMFeatures2Davg | Run percentage | 0.825222 |
| GLRLMFeatures2Davg | Grey level variance | 242.636 |
| GLRLMFeatures2Davg | Run length variance | 0.368043 |
| GLRLMFeatures2Davg | Run entropy | 422.683 |
| GLRLMFeatures2DDmrg | short run emphasis | 0.881272 |
| GLRLMFeatures2DDmrg | long runs emphasis | 195.728 |
| GLRLMFeatures2DDmrg | Low grey level run emphasis | 0.0012313 |
| GLRLMFeatures2DDmrg | High grey level run emphasis | 960.52 |
| GLRLMFeatures2DDmrg | Short run low grey level emphasis | 0.00112536 |
| GLRLMFeatures2DDmrg | Short run high grey level emphasis | 816.215 |
| GLRLMFeatures2DDmrg | Long run low grey level emphasis | 0.00204614 |
| GLRLMFeatures2DDmrg | Long run high grey level emphasis | 2152.12 |
| GLRLMFeatures2DDmrg | Grey level non uniformity | 134.364 |
| GLRLMFeatures2DDmrg | Grey level non uniformity normalized | 0.055778 |
| GLRLMFeatures2DDmrg | Run length non uniformity | 1778.56 |
| GLRLMFeatures2DDmrg | Run length non uniformity normalized | 0.738013 |
| GLRLMFeatures2DDmrg | Run percentage | 0.813049 |
| GLRLMFeatures2DDmrg | Grey level variance | 334.654 |
| GLRLMFeatures2DDmrg | Run length variance | 0.441533 |
| GLRLMFeatures2DDmrg | Run entropy | 502.357 |
| GLRLMFeatures2DWmrg | short run emphasis | 0.880544 |
| GLRLMFeatures2DWmrg | long runs emphasis | 186.999 |
| GLRLMFeatures2DWmrg | Low grey level run emphasis | 0.00141163 |
| GLRLMFeatures2DWmrg | High grey level run emphasis | 876.3 |
| GLRLMFeatures2DWmrg | Short run low grey level emphasis | 0.00127733 |
| GLRLMFeatures2DWmrg | Short run high grey level emphasis | 747.438 |
| GLRLMFeatures2DWmrg | Long run low grey level emphasis | 0.00227169 |
| GLRLMFeatures2DWmrg | Long run high grey level emphasis | 1887.21 |
| GLRLMFeatures2DWmrg | Grey level non uniformity | 381.528 |
| GLRLMFeatures2DWmrg | Grey level non uniformity normalized | 0.108427 |
| GLRLMFeatures2DWmrg | Run length non uniformity | 396.346 |
| GLRLMFeatures2DWmrg | Run length non uniformity normalized | 0.742205 |
| GLRLMFeatures2DWmrg | Run percentage | 0.825222 |
| GLRLMFeatures2DWmrg | Grey level variance | 242.885 |
| GLRLMFeatures2DWmrg | Run length variance | 0.377999 |
| GLRLMFeatures2DWmrg | Run entropy | 438.868 |
| GLRLMFeatures2Dvmrg | short run emphasis | 0.881551 |
| GLRLMFeatures2Dvmrg | long runs emphasis | 195.287 |
| GLRLMFeatures2Dvmrg | Low grey level run emphasis | 0.00123113 |
| GLRLMFeatures2Dvmrg | High grey level run emphasis | 960.653 |
| GLRLMFeatures2Dvmrg | Short run low grey level emphasis | 0.00112548 |
| GLRLMFeatures2Dvmrg | Short run high grey level emphasis | 816.662 |
| GLRLMFeatures2Dvmrg | Long run low grey level emphasis | 0.00204199 |
| GLRLMFeatures2Dvmrg | Long run high grey level emphasis | 2146.96 |
| GLRLMFeatures2Dvmrg | Grey level non uniformity | 536.784 |
| GLRLMFeatures2Dvmrg | Grey level non uniformity normalized | 0.0557234 |
| GLRLMFeatures2Dvmrg | Run length non uniformity | 7111.99 |
| GLRLMFeatures2Dvmrg | Run length non uniformity normalized | 0.738295 |
| GLRLMFeatures2Dvmrg | Run percentage | 0.813049 |
| GLRLMFeatures2Dvmrg | Grey level variance | 334.749 |
| GLRLMFeatures2Dvmrg | Run length variance | 0.440121 |
| GLRLMFeatures2Dvmrg | Run entropy | 50.429 |
| GLRLMFeatures3Davg | short run emphasis | 0.890554 |
| GLRLMFeatures3Davg | long runs emphasis | 18.125 |
| GLRLMFeatures3Davg | Low grey level run emphasis | 0.00122477 |
| GLRLMFeatures3Davg | High grey level run emphasis | 965.624 |
| GLRLMFeatures3Davg | Short run low grey level emphasis | 0.00112815 |
| GLRLMFeatures3Davg | Short run high grey level emphasis | 832.01 |
| GLRLMFeatures3Davg | Long run low grey level emphasis | 0.00191078 |
| GLRLMFeatures3Davg | Long run high grey level emphasis | 1980.75 |
| GLRLMFeatures3Davg | Grey level non uniformity | 140.331 |
| GLRLMFeatures3Davg | Grey level non uniformity normalized | 0.0568686 |
| GLRLMFeatures3Davg | Run length non uniformity | 1864.42 |
| GLRLMFeatures3Davg | Run length non uniformity normalized | 0.755181 |
| GLRLMFeatures3Davg | Run percentage | 0.832494 |
| GLRLMFeatures3Davg | Grey level variance | 334.218 |
| GLRLMFeatures3Davg | Run length variance | 0.363215 |
| GLRLMFeatures3Davg | Run entropy | 495.659 |
| GLRLMFeatures3Dmrg | short run emphasis | 0.891061 |
| GLRLMFeatures3Dmrg | long runs emphasis | 180.253 |
| GLRLMFeatures3Dmrg | Low grey level run emphasis | 0.0012243 |
| GLRLMFeatures3Dmrg | High grey level run emphasis | 966.009 |
| GLRLMFeatures3Dmrg | Short run low grey level emphasis | 0.00112816 |
| GLRLMFeatures3Dmrg | Short run high grey level emphasis | 833 |
| GLRLMFeatures3Dmrg | Long run low grey level emphasis | 0.00190182 |
| GLRLMFeatures3Dmrg | Long run high grey level emphasis | 1968.63 |
| GLRLMFeatures3Dmrg | Grey level non uniformity | 1820.18 |
| GLRLMFeatures3Dmrg | Grey level non uniformity normalized | 0.0567813 |
| GLRLMFeatures3Dmrg | Run length non uniformity | 24226.4 |
| GLRLMFeatures3Dmrg | Run length non uniformity normalized | 0.755754 |
| GLRLMFeatures3Dmrg | Run percentage | 0.832494 |
| GLRLMFeatures3Dmrg | Grey level variance | 334.574 |
| GLRLMFeatures3Dmrg | Run length variance | 0.359627 |
| GLRLMFeatures3Dmrg | Run entropy | 498.206 |
| GLSZMFeatures2Davg | small zone emphasis | 0.697846 |
| GLSZMFeatures2Davg | Large zone emphasis | 141.793 |
| GLSZMFeatures2Davg | Low grey level zone emphasis | 0.00153577 |
| GLSZMFeatures2Davg | High grey level zone emphasis | 785.09 |
| GLSZMFeatures2Davg | Small zone low grey level emphasis | 0.00106738 |
| GLSZMFeatures2Davg | Small zone high grey level emphasis | 532.626 |
| GLSZMFeatures2Davg | Large zone low grey level emphasis | 0.0129185 |
| GLSZMFeatures2Davg | Large zone high grey level emphasis | 17182.6 |
| GLSZMFeatures2Davg | Grey level non uniformity GLSZM | 510.213 |
| GLSZMFeatures2Davg | Grey level non uniformity normalized GLSZM | 0.0995354 |
| GLSZMFeatures2Davg | Zone size non uniformity | 461.667 |
| GLSZMFeatures2Davg | Zone size non uniformity normalized | 0.566007 |
| GLSZMFeatures2Davg | Zone percentage GLSZM | 0.527749 |
| GLSZMFeatures2Davg | Grey level variance GLSZM | 228.634 |
| GLSZMFeatures2Davg | Zone size variance | 102.695 |
| GLSZMFeatures2Davg | Zone size entropy | 458.415 |
| GLSZMFeatures2Dvmrg | small zone emphasis | 0.758871 |
| GLSZMFeatures2Dvmrg | Large zone emphasis | 161.294 |
| GLSZMFeatures2Dvmrg | Low grey level zone emphasis | 0.00138818 |
| GLSZMFeatures2Dvmrg | High grey level zone emphasis | 843.944 |
| GLSZMFeatures2Dvmrg | Small zone low grey level emphasis | 0.00111164 |
| GLSZMFeatures2Dvmrg | Small zone high grey level emphasis | 599.173 |
| GLSZMFeatures2Dvmrg | Large zone low grey level emphasis | 0.0134853 |
| GLSZMFeatures2Dvmrg | Large zone high grey level emphasis | 20265.6 |
| GLSZMFeatures2Dvmrg | Grey level non uniformity GLSZM | 759.582 |
| GLSZMFeatures2Dvmrg | Grey level non uniformity normalized GLSZM | 0.0496459 |
| GLSZMFeatures2Dvmrg | Zone size non uniformity | 817.043 |
| GLSZMFeatures2Dvmrg | Zone size non uniformity normalized | 0.534015 |
| GLSZMFeatures2Dvmrg | Zone percentage GLSZM | 0.516543 |
| GLSZMFeatures2Dvmrg | Grey level variance GLSZM | 298.673 |
| GLSZMFeatures2Dvmrg | Zone size variance | 123.815 |
| GLSZMFeatures2Dvmrg | Zone size entropy | 569.066 |
| GLSZMFeatures3D | small zone emphasis | 0.573178 |
| GLSZMFeatures3D | Large zone emphasis | 478.245 |
| GLSZMFeatures3D | Low grey level zone emphasis | 0.00162162 |
| GLSZMFeatures3D | High grey level zone emphasis | 709.354 |
| GLSZMFeatures3D | Small zone low grey level emphasis | 0.000989783 |
| GLSZMFeatures3D | Small zone high grey level emphasis | 380.36 |
| GLSZMFeatures3D | Large zone low grey level emphasis | 0.368054 |
| GLSZMFeatures3D | Large zone high grey level emphasis | 627205 |
| GLSZMFeatures3D | Grey level non uniformity GLSZM | 413.686 |
| GLSZMFeatures3D | Grey level non uniformity normalized GLSZM | 0.0569032 |
| GLSZMFeatures3D | Zone size non uniformity | 228.125 |
| GLSZMFeatures3D | Zone size non uniformity normalized | 0.31379 |
| GLSZMFeatures3D | Zone percentage GLSZM | 0.245442 |
| GLSZMFeatures3D | Grey level variance GLSZM | 230.144 |
| GLSZMFeatures3D | Zone size variance | 461.645 |
| GLSZMFeatures3D | Zone size entropy | 622.735 |
| ngtdmFeatures2avg | coarseness | 0.19264 |
| ngtdmFeatures2avg | contrast | 0.155963 |
| ngtdmFeatures2avg | busyness | 0.0974996 |
| ngtdmFeatures2avg | complexity | 177.544 |
| ngtdmFeatures2avg | strength | 123.208 |
| ngtdmFeatures2Dmrg | coarseness | 0.00557074 |
| ngtdmFeatures2Dmrg | contrast | 0.151125 |
| ngtdmFeatures2Dmrg | busyness | 0.257585 |
| ngtdmFeatures2Dmrg | complexity | 258.383 |
| ngtdmFeatures2Dmrg | strength | 121.335 |
| ngtdmFeatures3D | coarseness | 0.00481281 |
| ngtdmFeatures3D | contrast | 0.173229 |
| ngtdmFeatures3D | busyness | 0.298149 |
| ngtdmFeatures3D | complexity | 298.334 |
| ngtdmFeatures3D | strength | 105.853 |
| gldzmFeatures2Davg | small distance emphasis GLDZM | 0.534907 |
| gldzmFeatures2Davg | Large distance emphasis GLDZM | 556.724 |
| gldzmFeatures2Davg | Low grey level zone emphasis GLDZM | 0.00153577 |
| gldzmFeatures2Davg | High grey level zone emphasis GLDZM | 785.09 |
| gldzmFeatures2Davg | Small distance low grey level emphasis GLDZM | 0.00103212 |
| gldzmFeatures2Davg | Small distance high grey level emphasis GLDZM | 319.574 |
| gldzmFeatures2Davg | Large distance low grey level emphasis GLDZM | 0.0059731 |
| gldzmFeatures2Davg | Large distance high grey level emphasis GLDZM | 5800.57 |
| gldzmFeatures2Davg | Grey level non uniformity GLDZM | 510.213 |
| gldzmFeatures2Davg | Grey level non uniformity normalized GLDZM | 0.0995354 |
| gldzmFeatures2Davg | Zone distance non uniformity GLDZM | 243.188 |
| gldzmFeatures2Davg | Zone distance non uniformity normalized GLDZM | 0.360682 |
| gldzmFeatures2Davg | Zone percentage GLDZM | 0.527749 |
| gldzmFeatures2Davg | Grey level variance GLDZM | 156.406 |
| gldzmFeatures2Davg | Zone distance variance GLDZM | 124.169 |
| gldzmFeatures2Davg | Zone distance entropy GLDZM | 444.916 |
| gldzmFeatures2Dmrg | small distance emphasis GLDZM | 0.483739 |
| gldzmFeatures2Dmrg | Large distance emphasis GLDZM | 603.399 |
| gldzmFeatures2Dmrg | Low grey level zone emphasis GLDZM | 0.00138818 |
| gldzmFeatures2Dmrg | High grey level zone emphasis GLDZM | 843.944 |
| gldzmFeatures2Dmrg | Small distance low grey level emphasis GLDZM | 0.000845956 |
| gldzmFeatures2Dmrg | Small distance high grey level emphasis GLDZM | 314.105 |
| gldzmFeatures2Dmrg | Large distance low grey level emphasis GLDZM | 0.00614036 |
| gldzmFeatures2Dmrg | Large distance high grey level emphasis GLDZM | 6502.75 |
| gldzmFeatures2Dmrg | Grey level non uniformity GLDZM | 759.582 |
| gldzmFeatures2Dmrg | Grey level non uniformity normalized GLDZM | 0.0496459 |
| gldzmFeatures2Dmrg | Zone distance non uniformity GLDZM | 424.844 |
| gldzmFeatures2Dmrg | Zone distance non uniformity normalized GLDZM | 0.277676 |
| gldzmFeatures2Dmrg | Zone percentage GLDZM | 0.129136 |
| gldzmFeatures2Dmrg | Grey level variance GLDZM | 298.673 |
| gldzmFeatures2Dmrg | Zone distance variance GLDZM | 145.495 |
| gldzmFeatures2Dmrg | Zone distance entropy GLDZM | 579.286 |
| gldzmFeatures3D | small distance emphasis GLDZM | 0.753916 |
| gldzmFeatures3D | Large distance emphasis GLDZM | 224.759 |
| gldzmFeatures3D | Low grey level zone emphasis GLDZM | 0.00162162 |
| gldzmFeatures3D | High grey level zone emphasis GLDZM | 709.354 |
| gldzmFeatures3D | Small distance low grey level emphasis GLDZM | 0.00136272 |
| gldzmFeatures3D | Small distance high grey level emphasis GLDZM | 467.18 |
| gldzmFeatures3D | Large distance low grey level emphasis GLDZM | 0.00287922 |
| gldzmFeatures3D | Large distance high grey level emphasis GLDZM | 1996.81 |
| gldzmFeatures3D | Grey level non uniformity GLDZM | 413.686 |
| gldzmFeatures3D | Grey level non uniformity normalized GLDZM | 0.0569032 |
| gldzmFeatures3D | Zone distance non uniformity GLDZM | 392.29 |
| gldzmFeatures3D | Zone distance non uniformity normalized GLDZM | 0.539601 |
| gldzmFeatures3D | Zone percentage GLDZM | 0.245442 |
| gldzmFeatures3D | Grey level variance GLDZM | 230.144 |
| gldzmFeatures3D | Zone distance variance GLDZM | 0.36311 |
| gldzmFeatures3D | Zone distance entropy GLDZM | 47.874 |
| ngldmFeatures2Davg | Low dependence emphasis | 0.446507 |
| ngldmFeatures2Davg | High dependence emphasis | 82.616 |
| ngldmFeatures2Davg | Low grey level count emphasis | 0.00136321 |
| ngldmFeatures2Davg | High grey level count emphasis | 912.59 |
| ngldmFeatures2Davg | Low dependence low grey level emphasis | 0.000675647 |
| ngldmFeatures2Davg | Low dependence high grey level emphasis | 351.147 |
| ngldmFeatures2Davg | High dependence low grey level emphasis | 0.00834328 |
| ngldmFeatures2Davg | High dependence high grey level emphasis | 9464.61 |
| ngldmFeatures2Davg | Grey level non uniformity | 144.174 |
| ngldmFeatures2Davg | Grey level non uniformity normalized | 0.122497 |
| ngldmFeatures2Davg | Dependence count non uniformity | 423.287 |
| ngldmFeatures2Davg | Dependence count non uniformity normalized | 0.32947 |
| ngldmFeatures2Davg | Dependence count percentage | 1 |
| ngldmFeatures2Davg | Grey level variance | 233.739 |
| ngldmFeatures2Davg | Dependence count variance | 227.225 |
| ngldmFeatures2Davg | Dependence count entropy | 49.242 |
| ngldmFeatures2Davg | dependence Count Energy | 0.0736202 |
| ngldmFeatures2Dmrg | Low dependence emphasis | 0.459014 |
| ngldmFeatures2Dmrg | High dependence emphasis | 918.096 |
| ngldmFeatures2Dmrg | Low grey level count emphasis | 0.00116338 |
| ngldmFeatures2Dmrg | High grey level count emphasis | 1011.43 |
| ngldmFeatures2Dmrg | Low dependence low grey level emphasis | 0.000644325 |
| ngldmFeatures2Dmrg | Low dependence high grey level emphasis | 382.776 |
| ngldmFeatures2Dmrg | High dependence low grey level emphasis | 0.00793509 |
| ngldmFeatures2Dmrg | High dependence high grey level emphasis | 11340.4 |
| ngldmFeatures2Dmrg | Grey level non uniformity | 198.968 |
| ngldmFeatures2Dmrg | Grey level non uniformity normalized | 0.0671736 |
| ngldmFeatures2Dmrg | Dependence count non uniformity | 711.695 |
| ngldmFeatures2Dmrg | Dependence count non uniformity normalized | 0.240275 |
| ngldmFeatures2Dmrg | Dependence count percentage | 1 |
| ngldmFeatures2Dmrg | Grey level variance | 328.494 |
| ngldmFeatures2Dmrg | Dependence count variance | 295.288 |
| ngldmFeatures2Dmrg | Dependence count entropy | 608.763 |
| ngldmFeatures2Dmrg | dependence Count Energy | 0.0173102 |
| ngldmFeatures3Dmrg | Low dependence emphasis | 0.20902 |
| ngldmFeatures3Dmrg | High dependence emphasis | 488.123 |
| ngldmFeatures3Dmrg | Low grey level count emphasis | 0.00116338 |
| ngldmFeatures3Dmrg | High grey level count emphasis | 1011.43 |
| ngldmFeatures3Dmrg | Low dependence low grey level emphasis | 0.000332255 |
| ngldmFeatures3Dmrg | Low dependence high grey level emphasis | 153.484 |
| ngldmFeatures3Dmrg | High dependence low grey level emphasis | 0.0392949 |
| ngldmFeatures3Dmrg | High dependence high grey level emphasis | 62561.6 |
| ngldmFeatures3Dmrg | Grey level non uniformity | 198.968 |
| ngldmFeatures3Dmrg | Grey level non uniformity normalized | 0.0671736 |
| ngldmFeatures3Dmrg | Dependence count non uniformity | 341.585 |
| ngldmFeatures3Dmrg | Dependence count non uniformity normalized | 0.115322 |
| ngldmFeatures3Dmrg | Dependence count percentage | 1 |
| ngldmFeatures3Dmrg | Grey level variance | 328.494 |
| ngldmFeatures3Dmrg | Dependence count variance | 201.345 |
| ngldmFeatures3Dmrg | Dependence count entropy | 698.222 |
| ngldmFeatures3Dmrg | dependence Count Energy | 0.00935573 |
